# Urban color in public design: a review of spatial aesthetics and behavioral impact in Chinese and South Korean cities using structural equation modelling approaches

**DOI:** 10.3389/fpsyg.2026.1692740

**Published:** 2026-01-27

**Authors:** Hang Yu, Ruitong Liu, Raman Kumar, Sehijpal Singh, Rajender Kumar

**Affiliations:** 1 Space Design, School of Design, Chung-Ang University, Dongjak-gu, Seoul, Republic of Korea; 2 Department of Industrial Design, School of Design, Chung-Ang University, Dongjak-gu, Seoul, Republic of Korea; 3 Department of Mechanical and Production Engineering, Guru Nanak Dev Engineering College, Ludhiana, Punjab, India; 4 Jadara Research Center, Jadara University, Irbid, Jordan; 5 Department of Mechanical and Production Engineering, Guru Nanak Dev Engineering College, Ludhiana, Punjab, India; 6 Department of Mechanical Engineering, Graphic Era (Deemed to be University), Clement Town, Dehradun, India; 7 Centre for Research Impact and Outcome, Chitkara University Institute of Engineering and Technology, Chitkara University, Rajpura, Punjab, India; 8 Department of Mechanical Engineering, Chandigarh University, Gharuan, Mohali, Punjab, India

**Keywords:** behavioral perception, East Asian cities, public design, spatial aesthetics, structural equation modelling, urban color

## Abstract

Urban colour is increasingly recognised as a performative design variable influencing safety, thermal comfort, wayfinding, and energy demand, yet empirical evidence remains fragmented. This review synthesizes peer-reviewed studies using structural equation modelling (SEM) to relate objectively measured chromatic metrics to psychosocial and environmental constructs in urban contexts across China and South Korea. Focusing on exterior colour, SEM application, and model fit, 73 studies were systematically coded for metrics, constructs, quality, and contextual moderators. Three consistent causal pathways emerge: (1) mid-range colour complexity enhances perceived safety and comfort (median *β* ≈ 0.43); (2) dominant hue and chroma improve wayfinding and place identity (*β* ≈ 0.30); and (3) cool or reflective palettes reduce heat stress and annual building energy use by 2–4%. Integration of AI-driven image segmentation, extended reality (XR) user testing, and multi-level SEM frameworks has enabled more robust causal inference. Recent findings also emphasize the moderating effects of policy, density, and demographics. This review highlights how advanced spatial information technologies, digital twins, geo-computation, and real-time SEM dashboards can inform evidence-based strategies for enhancing the quality, safety, and comfort of urban living spaces. Collectively, these innovations offer transformative potential to optimize public design and foster sustainable, data-driven urban well-being.

## 1 Introduction

One of the most immediate sensory cues encountered in the urban environment, whether along streetscapes, public plazas, or skylines, is colour. From the vermilion gates of Seoul’s Gyeongbokgung Palace to the subdued greys of Wuhan’s Yangtze riverfront façades, chromatic systems function as carriers of cultural identity, mediators of thermal comfort, and facilitators of wayfinding. Over the past decade, research into urban colour has moved from the periphery of architectural aesthetics into the core of evidence-based urban design (Zhang and Li, 2013; Asarzadeh et al., 2020). With recent advances in large-scale data acquisition encompassing street-view imagery, unmanned aerial vehicle (UAV) footage, satellite-based spectral rasters, and physiological sensors, researchers now possess the capacity to quantify hue, saturation, and luminance across entire urban areas, and statistically relate these metrics to latent perceptual and behavioral constructs using structural equation modelling (SEM) (Yu et al., 2024; Wu et al., 2025).

This research landscape is particularly fertile within the contexts of China and South Korea. Despite the existence of official chromatic guidelines at both national and subnational levels intended to inform colour strategies in historic precincts and master-planned developments, rapid urbanisation and shifting citizen expectations have exposed significant gaps between regulatory palettes and lived chromatic experience (Chiu et al., 2024). Contemporary artificial intelligence (AI) methodologies, including semantic segmentation, self-organising maps, and attention-based change detection, now make it possible to systematically audit the chromatic composition of entire street networks for regulatory alignment and perceptual coherence, at scale and in near real-time (Song and Xiao, 2025). Concurrently, extended-reality (XR) environments allow urban designers to simulate and prototype colour interventions, capturing neurophysiological responses (including electroencephalographic activity) before implementation (Bustos-Lopez et al., 2024; Baradaran Rahimi, 2025). Against this confluence of pressing policy needs, accessible technological platforms, and maturing analytic methods, East Asia emerges as a dynamic testbed for advancing urban chromatic practice (Hawken and Isendahl, 2025).

Nonetheless, the extant literature remains fragmented. The majority of studies are cross-sectional in design, rely on single-source data inputs, and often neglect moderating variables such as policy stringency or demographic inequities. Only a limited subset of research integrates objective environmental indicators, such as sky-view factor or fine particulate concentration, with subjective colour appraisals, and fewer still enact a complete analytical feedback loop in which SEM-derived insights inform adaptive or interactive design systems (Chen and Hanlon, 2025). As urban colour gains traction within public policy from Shanghai’s “Chromatic Excellence” programme to Busan’s recent façade regulation updates, there is an urgent need for an integrated evidence base (Márquez, 2024). Such a foundation must synthesise perceptual, environmental, and equity considerations to guide both regulatory frameworks and urban design practice.

### 1.1 Background and importance of urban color in public design

The color of a city is often an underappreciated yet significant element that shapes the visual character, experiential quality, and overall ambiance of urban life. It contributes to urban identity, facilitates wayfinding, and influences the psychological well-being of residents (Zhang and Li, 2013). In cities such as Wuhan, color zoning strategies that divide urban areas into visually distinct partitions, such as central and transitional zones, have been employed to preserve urban character and enhance visual coherence. However, early implementations of color-coding, such as those in the historic center of Tehran, have proven to be limited in scope, primarily due to a lack of consideration for figure-ground perception and spatial legibility (Asarzadeh et al., 2020). Consequently, the inflexibility inherent in such schemes emerges as a principal challenge to maintaining consistent and effective color strategies in practice.

With the advent of smart city initiatives and advancements in street-level image analytics, recent scholarship has increasingly adopted data-driven approaches to assess urban color. For instance, Yu et al. (2024) demonstrated how attributes such as color complexity and coordination influence emotional responses, including perceived vitality, safety, and discomfort. Likewise, Wu et al. (2025) used ensemble learning techniques to evaluate color alongside other visual factors.

These findings emphasize a growing recognition that urban color serves functions beyond mere decoration; it is a fundamental factor shaping spatial aesthetics, behavioral responses, and principles of sustainable urban design. When employed strategically, color enhances visual continuity and fosters emotional engagement within public spaces.

### 1.2 Research objectives and scope

The primary objective of this study is to systematically review and synthesize existing scholarly research on the role of urban color in public design and its behavioral and psychological impacts, with a particular emphasis on studies using SEM. Focusing on urban contexts in China and South Korea, the review aims to consolidate fragmented empirical evidence and clarify how color-related design attributes influence perception, emotion, and behavior in public spaces.

While the empirical focus of this review is on China and South Korea, the study is positioned within a broader global discourse on urban color in public design. These two countries are selected not as regionally exceptional cases but as analytically representative contexts in which rapid urbanization, high-density development, and decisive policy intervention have accelerated the formalization of urban color strategies. China exemplifies large-scale, state-led urban transformation with emerging national and municipal color guidelines. In contrast, South Korea represents a design- and citizen-oriented planning paradigm characterized by participatory governance and perceptual evaluation. Together, these contrasting yet complementary contexts provide a robust comparative framework for examining how urban color influences behavioral and psychological outcomes. The methodological insights derived, particularly regarding the application of SEM to color perception, safety, and wayfinding, are transferable to diverse urban settings beyond East Asia, including European, North American, and rapidly urbanizing cities in the Global South. As such, the review contributes not only to East Asian urban studies but also to the global understanding of evidence-based color design in public spaces. This review pursues the following objectives:

- To examine key theoretical perspectives and conceptual frameworks underpinning urban color research in public design contexts.
- To synthesize empirical findings on the psychological and behavioral effects of urban color, including perceived safety, comfort, identity, and wayfinding behavior.
- To critically analyze how SEM has been applied to model latent constructs and causal pathways in urban color research.
- To identify methodological limitations, research gaps, and emerging directions, including opportunities for integrating AI-based and geospatial color analysis within future SEM studies.

This review examines the role of color in public interiors and semi-interiors in urban environments, such as metro stations, transport hubs, hospitals, and other publicly accessible enclosed spaces, where color is critical to perceived safety, wayfinding, comfort, and behavioral responses. The review focuses on peer-reviewed English-language journal articles, with an emphasis on exterior urban environments and public spaces. Rather than proposing prescriptive design solutions, the study emphasizes analytical synthesis and methodological insight, providing a structured knowledge base to inform future empirical and comparative research in urban color studies.

## 2 Methodology

Given that urban colour research spans multiple disciplinary domains including urban design, environmental psychology, landscape planning, and computational modelling a narrative scoping review was adopted as the primary methodological framework. This approach is appropriate for conceptually broad yet methodologically structured fields, enabling transparent synthesis while maintaining replicability.

A systematic literature search was conducted across major abstracting and indexing databases commonly used in urban design and environmental-behavior research, including Scopus, Web of Science Core Collection, CNKI (for Chinese-language journals), Google Scholar, IEEE Access, and RISS (for Korean-language journals).

Core chromatic search terms (e.g., *urban colour*, *city color design*, *façade chromatics*, *streetscape colour*) were systematically combined with thematic keywords reflecting the analytical focus of this review (e.g., *public design*, *behavioral influence*, *structural equation modelling*, *machine learning*). Boolean operators and wildcard logic were applied to capture British and American spelling variants and relevant synonyms. Language restrictions were used only during the abstract-screening stage to avoid premature exclusion of bilingual or regionally indexed publications.

**Figure 1:**
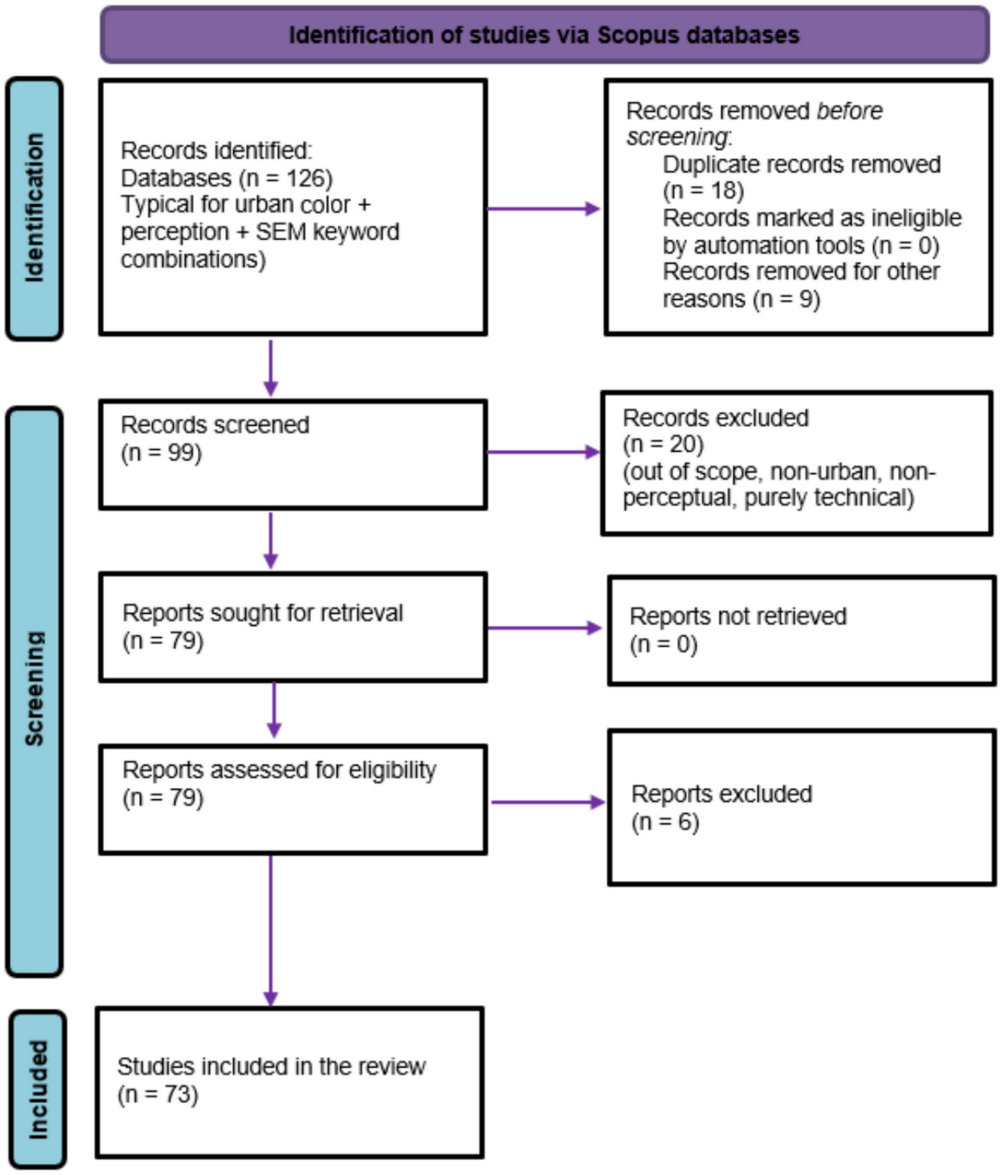
PRISMA flow diagram of literature search and study selection.

Figure 1 illustrates the PRISMA-based screening and selection process employed in this study, detailing database identification, duplicate removal, eligibility assessment, and the final inclusion of 73 studies. The review followed a PRISMA-compliant multi-stage screening process to ensure methodological transparency and reproducibility. In total, 126 records were initially identified through database searches. After removing 18 duplicate records and nine records excluded for non-article document types, 99 records proceeded to title and abstract screening.

Following this step, 20 records were excluded for misalignment with the review’s thematic scope, leaving 79 full-text articles for eligibility assessment. Of these, six articles were excluded after full-text review because they lacked a clear urban-perceptual focus or a latent-variable analytical structure. Eventually, 73 studies met all inclusion criteria and were included in the final synthesis.

The literature identification and screening process was guided by the PRISMA (Preferred Reporting Items for Systematic Reviews and Meta-Analyses) framework (Kaur et al., 2025; Wang H. et al., 2025). Records retrieved from multiple databases were first consolidated and de-duplicated, then screened for titles and abstracts to exclude studies outside the scope of exterior urban colour and public design. A full-text eligibility assessment was then conducted using predefined inclusion and exclusion criteria, with reasons for exclusion documented at each stage. This structured workflow ensured transparency, reduced selection bias, and enabled systematic tracking of the review process (Page et al., 2022).

Studies were included if they met all of the following conditions:

- Empirical scope: Reporting primary data or secondary quantitative analyses addressing exterior colour or lighting in public-realm environments (e.g., building façades, streetscapes, parks, plazas).
- Geographic relevance: Incorporating at least one Chinese or South Korean case study, or advancing transferable theoretical frameworks empirically tested using East Asian urban data.
- Methodological rigor: Applying structural equation modelling (covariance-based SEM, PLS-SEM, multilevel SEM) or equivalent latent-variable/path-analytic techniques, with reported goodness-of-fit or model validation statistics.
- Language accessibility: To ensure analytical consistency, studies were required to provide English-language titles and abstracts, regardless of the language of the full text. This criterion enabled the inclusion of relevant Chinese and Korean-language publications indexed in CNKI and RISS, while maintaining transparent and replicable screening procedures.

Studies were excluded if they met any of the following conditions:

- Focused exclusively on interior colour applications (e.g., classrooms, residential interiors) or product/fashion chromatics.
- Relied solely on qualitative methods without latent-variable modelling or structural/path analysis.
- Were non-peer-reviewed materials, including editorials, commentaries, book chapters, patents, theses, exhibition catalogues, or magazine articles.
- Addressed non-urban contexts (e.g., rural landscapes, protected natural areas) or were limited to purely physical parameters (e.g., albedo or radiative properties) without behavioral or perceptual constructs.
- Consisted of duplicate records, irretrievable full texts, or publications that had been retracted or superseded by later versions.

## 3 Results

Following PRISMA-guided screening and eligibility assessment, a total of 73 peer-reviewed studies were retained for the final qualitative synthesis, as detailed in the PRISMA flow diagram and listed in the Supplementary Materials Excel file. The temporal distribution of publications reveals a marked growth after 2022, with the highest concentration of studies published between 2023 and 2025, indicating a rapid increase in scholarly interest in urban color, visual perception, and public design. In terms of authorship characteristics, the literature is dominated by multi-author and interdisciplinary research teams, primarily affiliated with institutions in East Asia (China), followed by Europe and Australia, reflecting strong regional engagement in data-driven urban design and environmental psychology research. Methodologically, earlier studies were largely qualitative and theory-driven. In contrast, recent publications increasingly employ quantitative analytics, machine learning, street-view big data, virtual reality experiments, and physiological measurements, demonstrating a clear methodological evolution. The PRISMA-based results confirm that the reviewed studies are not isolated contributions but form a coherent and expanding research domain, with strong thematic convergence on human-centered urban color systems, perceptual comfort, and evidence-based public design strategies.

### 3.1 Urban color as a component of public design

**Figure 2:**
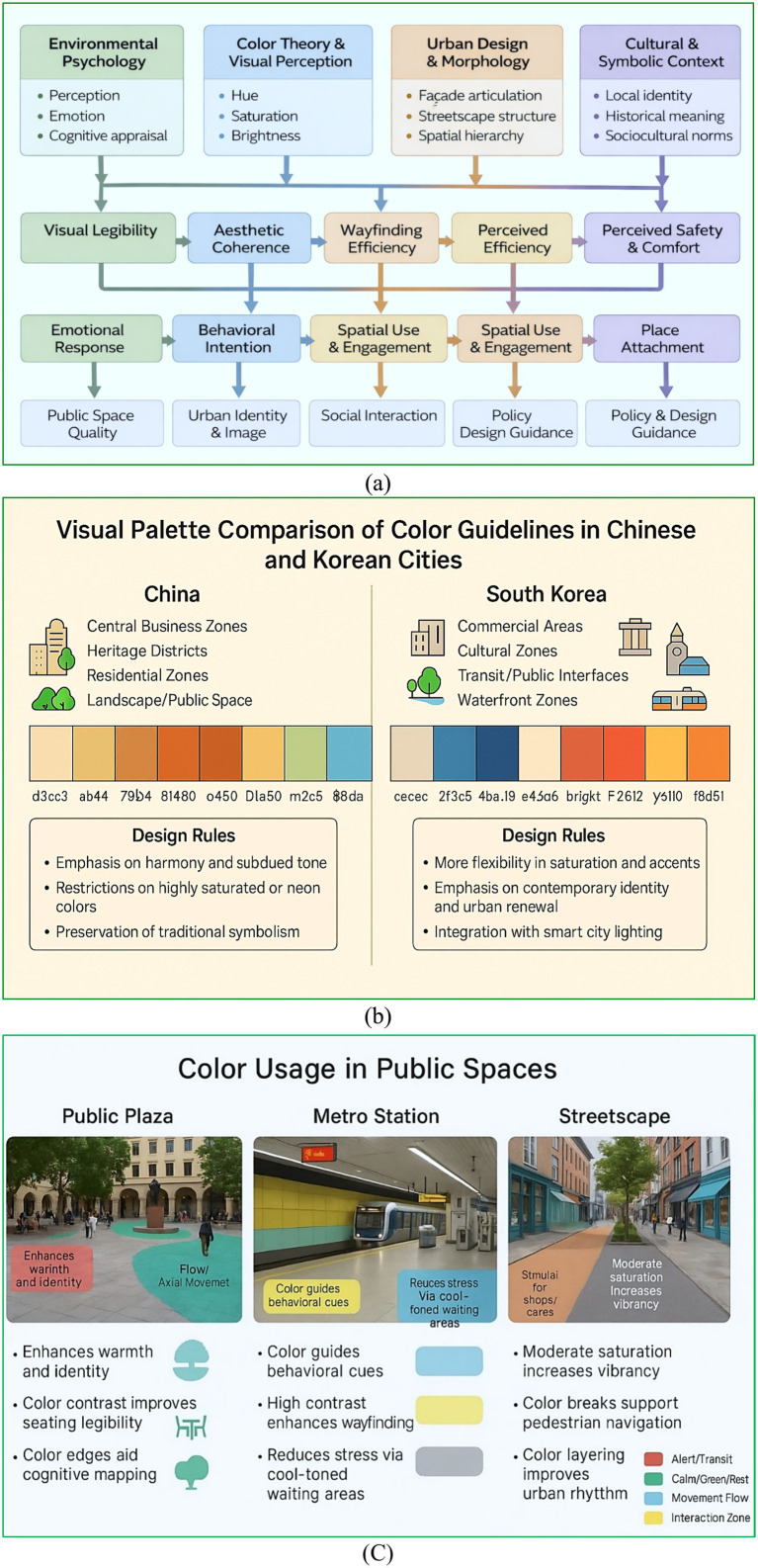
**(a)** Example of color usage in a public interior urban environment (metro station), illustrating directional guidance, spatial legibility, and perceived safety through chromatic cues **(a)** Theoretical foundations of urban color, **(b)** Visual palette comparison of color guidelines in Chinese and Korean cities, **(c)** Color usage in public spaces.

Urban color is examined across multiple dimensions: its role in spatial perception, its contribution to the creation of “atmospheres” for assessing emotional well-being, and its function in articulating the identity of public spaces. Color operates within theoretical frameworks encompassing psychology, semiotics, and aesthetics, as evidenced by its practical application through regional design codes in cities across China and South Korea (Figures 2a–c). Urban color is far from a superficial element; rather, it is deeply interwoven with the principles of environmental form and the evolving dynamics of the public realm. The design of metro station entrances incorporates regulation of façade color, material, and permeability to manage visual impact and maintain spatial continuity (Li et al., 2023). Similarly, urban parks characterized by plant-based landscapes, particularly during autumn, rely on the interplay of warm color harmonies and warm-cool contrasts to enhance perceived aesthetic value (Luo Y. et al., 2023).

Color serves as a symbolic language that influences the development of identity and architectural form, especially in heritage-rich urban contexts such as Brașov, Romania, where the built environment is imbued with cultural meaning (Petre-Spiru, 2024). Color planning acts as a mediator between form and function, as seen in the integration of green-toned ecological corridors that promote biodiversity (Hails and Kavanagh, 2013), as well as in attention-guiding mechanisms in urban scenes that interpret color perception through brainwave analysis (Wang L. et al., 2025). Large-scale epidemiological evidence demonstrates that higher exposure to urban greenness, measured through NDVI, is significantly associated with lower odds of major depressive disorders, particularly among socio-economically vulnerable populations.

These findings position green color exposure as a core component of public design, linking chromatic qualities of urban environments to measurable psychological and mental-health outcomes (Sarkar et al., 2018). (Li et al., 2025) demonstrated that the perceptual quality of neighborhood streets shaped by spatial interfaces, sidewalk structures, and human-centered design plays a decisive role in supporting leisure behavior and social interaction. Their framework, grounded in environmental psychology, implicitly reinforces urban color as a critical component of public design by enhancing visual comfort, spatial legibility, and emotional engagement within street-level leisure spaces. Consequently, the role of color in urban environments extends beyond visual aesthetics, encompassing behavioral, symbolic, and ecological dimensions.

#### 3.1.1 Definitions and theoretical foundations

Urban color, as a spatial and perceptual design construct, is embedded within a multiplicity of theoretical paradigms, each addressing distinct dimensions of color, such as roadway elements, through cognitive, emotional, cultural, and environmental lenses. A recent advancement in this domain is the development of a novel urban streetscape emotion quantification model: the Color Emotion Perception framework, incorporating K-Means, SegNet, and Support Vector Machine algorithms (CEP-KASS). Drawing on the Moon–Spencer Color Coordination Theory, this model emphasizes visual harmony through the continuity of warm tones, particularly in historic urban districts.

In a similar vein, the Cultural-Symbolic Design Theory conceptualizes urban color as a bearer of local identity and semiotic meaning, thereby serving as a communicative medium from a cultural identity perspective. Concurrently, AI-driven algorithms, such as the Environmental Color Enhancement model, employ deep learning methods to recalibrate color gradients across public and residential spaces. Furthermore, Interactive Genetic Algorithms simulate color vision as an evolutionary process, wherein psychophysical variable reproduction interacts dynamically with environmental asset presentation, thereby facilitating adaptive color design in marine urban contexts, as illustrated in Table 1.

**Table 1:** Theoretical approaches and definitions of urban color.

Theory/framework	Key concepts	Application area	Ref.
CEP-KASS (color emotion perception with K-Means, SegNet, SVM)	Machine learning-based perception modeling, visual-emotion mapping, color brightness vs. safety and vitality, perception quantification	Urban streetscape color planning (Tianjin, Xuzhou)	Hu et al. (2023), Chen et al. (2025)
Moon–Spencer color coordination theory	Objective-subjective harmony assessment, warm tone harmony, and color continuity in historic zones	Coastal historic districts (Mojiko, Japan)	Lyu et al. (2025)
Cultural-symbolic design theory	Color as a semiotic and cultural construct, urban material translation into design palettes, and urban legibility	Place-making, road paint, and tweed fabric reinterpretation	Mottram and Jefferies (2013)
Environmental color enhancement via deep learning	Digital quantization of color layers, AI-driven adjustment of visual gradation, and artistic focus	Environmental art design, residential public spaces	Weiwei and Bo (2024)
Interactive	Color-human psychology-environment interaction, adaptive aesthetic generation, color behavior modeling	Marine cities (Zhuhai), urban landscape, and well-being	Zhang and Kim (2023)

#### 3.1.2 Cultural symbolism and color perception

Colour conveys multifaceted cultural meanings through its spatial, emotional, and symbolic communicative functions across diverse cultural contexts. In Kazakh and Turkish toponyms, colour terminology is linked not only to geographical orientation but also to sacred symbolism (Abdrakhynova et al., 2025). Similarly, Indigenous traditions in North America ascribe cosmological and ritual significance to colour (Derzhavina et al., 2020). Linguistic and metaphorical expressions, such as the use of the colour red across Germanic and Tatar languages, further reveal culturally mediated worldviews (Mukhamadiarova et al., 2016). From traditional dyeing practices in Dong brocade (Hu and Junior, 2024) to contemporary approaches in art evaluation (Spee et al., 2024).

#### 3.1.3 Color guidelines in Chinese and South Korean urban planning

Urban colour references in China and South Korea reflect divergent approaches to chromatic governance, as outlined in Table 2. In China, data-driven models such as K-means cluster analysis and perceptual surveys have been employed in the design of urban parks and streetscapes to enhance both urban identity and resident well-being (Feng et al., 2024; Hao et al., 2024). A growing number of policy initiatives now incorporate considerations of surrounding ecological conditions and the emotional responses of residents (Qian, 2015). In contrast, South Korean frameworks tend to emphasize regulatory uniformity. Nevertheless, tensions persist between the centralized, top-down approach of governmental authorities and the diverse aesthetic preferences of citizens, as illustrated in the case of Busan (Wen et al., 2023). Additionally, environmental, infrastructural, and smart city policy measures indirectly shape urban colour schemes in both national contexts (Guttikunda et al., 2003; Lim and Taeihagh, 2018).

**Table 2:** National color guidelines in urban planning: China vs. South Korea.

Country	Policy name/concept	Issuing body	Color rules/palettes	Application areas	Ref.
China	Urban park color quantification	Urban landscape research (people’s park, Huanhuaxi park)	Medium saturation/high brightness for architecture; low brightness for pavements; seasonal plant color variation using K-means and SD methods	Urban Park color planning and public perception	Feng et al. (2024)
China	Air pollution and emission zones	Urban environmental policy planners	Emphasis on high-contrast warning zones (e.g., sulfur pollution alerts); lack of unified chromatic scheme	Emission-sensitive megacities (Shanghai, Chongqing)	Guttikunda et al. (2003)
China	Street greening and well-being	Local urban planning depts (Hangzhou)	Emphasis on green view index, plant diversity, color richness to improve emotional responses; seasonal flowering tree palettes	Street design, wellness-focused urban renewal	Hao et al. (2024)
South Korea	Land use and stream quality (environmental color planning)	Yeongsan river watershed committee	Focus on industrial/agricultural land use areas; indirect influence on urban color zoning through pollution source control	Environmental zoning, water buffer landscape	Kang et al. (2010)
South Korea	City color preferences vs. guidelines	Busan city planning authority	Tension between personal color preferences (chroma, hue) and government-imposed color standards; calls for bottom-up planning	Urban regeneration, participatory color planning	Wen et al. (2023)

### 3.2 Behavioral and psychological impact of urban color

**Figure 3:**
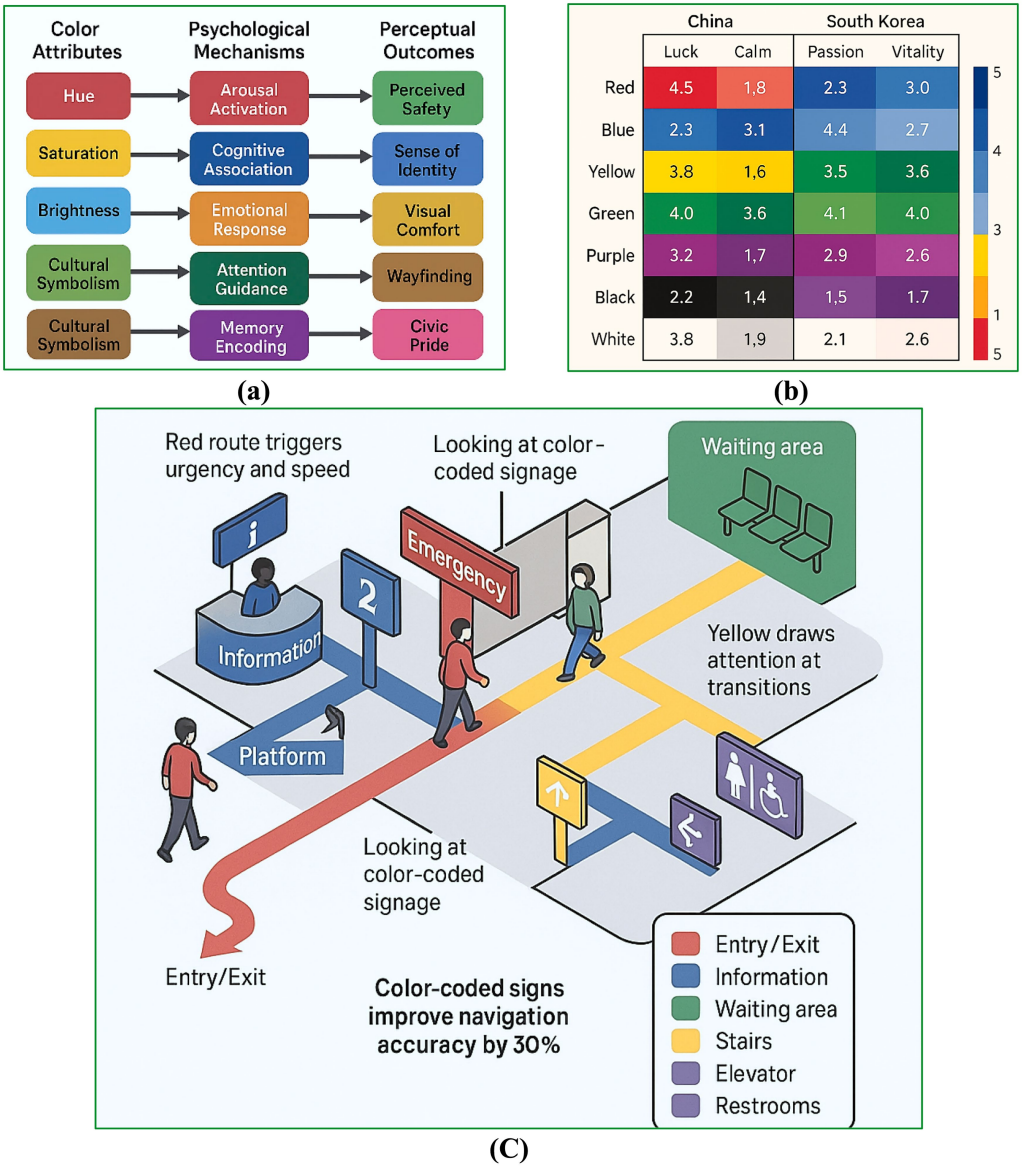
Conceptual illustration of psychological pathways linking color attributes to perception in public interior environments, including safety, comfort, and spatial identity: **(a)** Psychological pathways from color to perception, **(b)** emotion–color mapping across cultures, **(c)** urban color and wayfinding behavior in public space.

Various dimensions of urban colour significantly influence psychological responses, emotional well-being, and wayfinding behaviors. Colour operates through multiple perceptual pathways, shaping sensations of safety, identity, and comfort (Figure 3A). These perceptual processes are mediated by both instinctual reactions and culturally embedded meanings. For instance, the colour red may convey urgency in Chinese contexts, while it signifies passion in Korean settings; conversely, blue is often interpreted as a symbol of trust and calm in both cultures (Figure 3B). Such culturally specific mappings are instrumental in urban design and related disciplines, enabling alignment between intended emotional responses and the designed environment (Halarewicz, 2017; Zhang and Kim, 2023). Psychological states play a critical mediating role in shaping environmental perception. Empirical evidence from high-density subtropical cities indicates that stress, depression, and emotional states significantly narrow acceptable comfort ranges and alter thermal perception, even under identical physical conditions (Lin and Zhang, 2025). Perceived experience constructs are routinely modelled as latent variables predicting behavioral intention in applied settings (Zia and Alotaibi, 2024). These findings strengthen the theoretical rationale for modelling emotional and psychological variables as latent mediators in SEM-based urban colour studies, particularly when examining comfort, safety, and well-being outcomes. Zhang et al. (2024) showed that a systematically constructed urban color system, grounded in environmental color analysis, significantly improves visual coherence and place identity in historic urban areas. Their findings highlight urban color as a strategic instrument for public design and visual governance.

Colour further contributes to spatial orientation. The deliberate application of chromatic cues, such as yellow to indicate caution or green to define movement pathways, serves to reduce user confusion and stress by minimising perceptual visual noise and guiding users through complex spatial configurations, as observed in metro stations and healthcare facilities (Figure 3C). These design elements foster cognitive clarity and enhance psychological safety (Chen et al., 2024; Hilal and Mudhee, 2024).

Moreover, empirical studies suggest that highly saturated and intense colours can positively influence psychological resilience, a phenomenon particularly pertinent in high-stress or post-trauma urban scenarios (Alejandra Acuña et al., 2022; Finucane et al., 2022). Emotionally resonant palettes, along with culturally attuned chromatic selections within urban landscapes, have been shown to promote well-being, alleviate stress, and offer restorative environmental experiences (Qureshi and Rachid, 2022; Zhuang, 2025).

#### 3.2.1 Perceived safety, comfort, and identity

One of the primary ways in which colour shapes the urban experience is by fostering a sense of safety and belonging among residents. Previous studies suggest that warm colour tones and well-illuminated spaces, particularly those with uniform lighting sources, contribute to a heightened perception of safety and reduced anxiety in public environments (Peña-García et al., 2015; Maniee et al., 2025). Likewise, the presence of natural colours in recreational areas enhances visual comfort and supports the development of place attachment (Polat and Akay, 2015; Monteiro et al., 2016). Peters and D'Penna (2020) advocated for the application of biophilic colour strategies in educational and communal settings, as these not only reinforce identity but also restore cognitive and emotional well-being. Zhang et al. (2022) further note that seasonal variations in thermal-visual comfort, as well as behavior related to perceived comfort, are closely tied to the brightness of tree canopy colours.

#### 3.2.2 Emotional responses and cognitive mapping

The quality of cognitive mapping and the ease with which urban environments are interpreted often stem from emotional reactions to visual attributes, most notably colour. Geng et al. (2017) demonstrate that subjective perception of colour can influence motivations for green mobility, highlighting the role of colour-coded environments in travel behavior. Qi et al. (2025) employed machine learning methods, specifically Estimates of Sole Proportions (ESP), to quantify subjective emotional responses to longitudinal gradients in streetscape colour complexity, revealing that greater chromatic coordination elicits more positive emotional states.

#### 3.2.3 Wayfinding and social interaction through colour

Colour also functions as a foundational component of urban systems for intuitive wayfinding and social cohesion. Maniee et al. (2025) assert that lighting colour temperature and spatial distribution directly influence directional confidence and social visibility at night, thereby affecting patterns of movement and behavior. Garshasbi and Santamouris (2019) explained that thermochromic materials provide visual feedback on environmental conditions, especially in spaces where achieving or assessing thermal comfort is challenging. Xie et al. (2019) explored the application of high-reflectivity coatings to minimise glare and improve visual comfort in densely populated public transit environments. Table 3 depicts behavioral responses to urban color.

**Table 3:** Behavioral responses to urban color.

Study	Method	Variables measured	Key findings	Ref.
Thermochromic materials and energy	Review	Thermal comfort, energy use	Thermochromic coatings reduce energy use but need durability enhancement	Garshasbi and Santamouris (2019)
Urban heat prediction (Sylhet)	Remote sensing, ML prediction (ANN)	Urban heat, LULC change	Urban expansion increases thermal stress white roofs recommended	Kafy et al. (2022)
VR lighting and well-being	VR experiment, likert scale	Lighting comfort, Security	5,000 K optimal for comfort and security; 7,000 K for brightness	Maniee et al. (2025)
Street color and emotions	Image analysis, ML (FCN, RF)	Emotion, Color complexity	Color complexity 0.86 optimizes beauty; red/yellow = vibrant	Qi et al. (2025)
Food labels and behavior	Focus groups	Label influence, Food choice	Octagonal warning labels are most effective behaviorally	Taillie et al. (2024)
Flower colors and MSVM emotions	Online survey, semantic differential	Emotions, Landscape/flower color	Tropical landscapes are viewed more negatively by MSVMs	Thomas et al. (2024)
Youth civic engagement	Interviews and surveys (youth centers)	Civic empowerment, Disempowerment	Civic action linked to emotional empowerment in adverse settings	Wray-Lake and Abrams (2020)
Cool pavement coatings	Lab tests, multi-index evaluation	Thermal comfort, Coating performance	Optimal pigment ratio improves reflectance and pavement performance	Xie et al. (2019)
Seasonal tree characteristics	Meteorological and perception survey	Thermal and visual comfort	Leaf traits, especially L*, improve visual/thermal comfort	Zhang et al. (2022)

### 3.3 Structural equation modelling (SEM) in urban-colour research

SEM is increasingly adopted in urban color research due to its capacity to integrate perceptual, behavioral, and contextual variables within a unified analytical framework. Unlike conventional statistical approaches that examine observed variables in isolation, SEM enables the explicit modeling of latent psychological constructs such as perceived safety, comfort, satisfaction, and place identity while simultaneously assessing their measurement validity and structural interrelationships (Mitra et al., 2024). This capability is particularly relevant for urban color studies, where subjective perception often mediates the relationship between chromatic design elements and observable behavioral or experiential outcomes. Recent urban studies increasingly combine machine learning, spatial analytics, and behavioral modelling to capture complex, non-linear urban dynamics. For example, machine-learning-based analyses of intercity systems demonstrate how hierarchical urban structures and policy interventions generate delayed and asymmetric spatial effects, highlighting the value of integrative analytical frameworks capable of modelling indirect and time-lagged relationships (Luo J. et al., 2023). Such methodological advances support the growing adoption of SEM in urban colour research, where latent perceptual constructs and contextual moderators interact in similarly complex ways.

SEM provides a robust methodological basis for analyzing complex urban design phenomena across diverse cultural, policy, and demographic contexts. By incorporating demographic moderators, environmental attributes, and policy-related variables, SEM facilitates comparative and multi-level interpretations that are essential for evidence-based public design and planning guidelines. The increasing application of SEM in recent urban color studies reflects a broader methodological shift toward theory-driven, empirically testable models that can bridge visual design decisions, psychological responses, and social behavior. To enhance the coherence and analytical focus of the manuscript, detailed descriptive methodological content is presented in the Supplementary Materials.

More than a mere aesthetic overlay, colour significantly influences a range of perceptual and behavioral outcomes in urban contexts. It modifies perceived safety during nighttime travel (Peña-García et al., 2015), mediates thermal comfort beneath urban tree canopies (Zhang et al., 2022), guides nutritional choices via front-of-pack labelling (Taillie et al., 2024), symbolically reinforces civic identity among youth populations (Wray-Lake and Abrams, 2020). As these effects often occur concurrently and, in many cases, through indirect mechanisms such as mediation and moderation, SEM has emerged as a preferred analytical framework in urban colour research. SEM enables the examination of latent constructs (e.g., perceived sense of security), complex mediation chains (e.g., colour → comfort → behavior), and allows for theory testing, indirect effect estimation, and model comparison within a single, cohesive modelling architecture (Unlu et al., 2017).

#### 3.3.1 Data architecture for SEM

Raw chromatic data, specifically hue, saturation, and luminance, have increasingly become standardised variables in urban design studies employing SEM. SEM enables researchers to examine both direct and indirect relationships between visual attributes and latent constructs such as aesthetic perception and perceived security. A typical analytical framework comprises three principal components: the use of reliable, multi-item psychometric scales, testing for measurement invariance across groups, estimating structural paths, and evaluating the overall model fit.

- Observed Indicators

Chromatic characteristics such as hue angle, saturation, and luminance are typically derived from street-view imagery or façade reflectance spectra (Xie et al., 2019; Qi et al., 2025). Contextual covariates may include variables such as tree canopy fraction, traffic volume, land-use heterogeneity, and pollution indices (Manton et al., 2016; Garshasbi and Santamouris, 2019; Kafy et al., 2022). Self-reported measures, commonly rated on 5- or 7-point Likert scales, encompass perceptual domains including safety, comfort, visual quality, motivational response, and place identity (Polat and Akay, 2015; Maniee et al., 2025).

- Latent Constructs and Reliability

Frequently employed latent constructs include Perceived Safety, Wayfinding Ease, Thermal Comfort, Visual Preference, and Civic Empowerment. Shooshtarian and Ridley (2016) recommend that Confirmatory Factor Analysis (CFA) results demonstrate standardised factor loadings ≥ 0.60, Composite Reliability (CR) values exceeding 0.70, and Average Variance Extracted (AVE) exceeding 0.50 to ensure acceptable construct validity and internal consistency.

The most recent colour-focused SEM studies exhibit considerable diversity in both thematic scope and methodological approaches (Table 4). These investigations span various geographical and cultural contexts, including Iberian, Chinese, and Irish urban environments and, in certain instances, incorporate immersive virtual-reality (VR) streetscapes.

**Table 4:** Key variables in recent colour–SEM studies.

Short title	Setting	n	Colour metric	Psychosocial latent	Software	Ref.
Public Lighting and Safety	Granada (Spain)	275 pedestrians	Illuminance uniformity and CCT	Safety, well-being	AMOS	Peña-García et al. (2015)
Green-travel motivation	Nanjing (China)	1,244 residents	“Green/Red/Grey” travel typology	Environmental motivation	Mplus	Geng et al. (2017)
Cycling-risk mental maps	Galway (Ireland)	104 cyclists	Road-section colour codes	Perceived cycling risk	AMOS	Manton et al. (2016)
VR street lighting	Immersive VR streets	40 participants	3 CCT levels (3000–7,000 K)	Security, directional clarity	SmartPLS	Maniee et al. (2025)
Street colour and emotion	Xi’an (China)	1.2 M street-view images	Colour complexity and coordination	Pleasure, vitality	semopy	Qi et al. (2025)

Sample sizes vary considerably across these studies, ranging from small-scale VR experiments with approximately 40 participants to large-scale analyses involving over 1.2 million street-view images. This variation attests to the scalability of SEM, whether implemented using covariance-based estimators (e.g., AMOS, Mplus) or variance-based techniques (e.g., SmartPLS, *semopy*). These case studies underscore the utility of chromatic variables as either first-order predictors or mediating factors within broader psychosocial models. They also highlight SEM’s robustness across diverse urban morphologies and heterogeneous data structures.

Within urban colour studies, four latent constructs recur with notable frequency: Aesthetic Perception, Thermal/Visual Comfort, Perceived Safety, and Place Identity. These constructs are operationalised using five- or seven-point Likert scale items derived from validated instruments or developed through targeted piloting procedures. Qi et al. (2025) assessed the constructs of Pleasure and Vitality through semantic differential scales using adjective pairs such as “dull–lively” and “sad–cheerful.” Similarly, Maniee et al. (2025), in their overview of the external validity dataset, measured Security via both subjective fear of crime and objective environmental attributes (e.g., number of street crossings during virtual reality wayfinding tasks). Table 5 summarises the most frequently utilised constructs and their corresponding indicators, including psychometric reliability coefficients (Cronbach’s alpha, Composite Reliability).

**Table 5:** SEM constructs and measurement variables in urban-colour research.

Construct	Typical indicators (examples)	Scale type	Reliability (*α*)	Ref.
Aesthetic perception	Pleasure, Vitality, Novelty	5-pt SD	0.88	Qi et al. (2025)
Perceived safety	“I feel secure here at night.”“Lighting prevents crime.”	7-pt Likert	0.77	Maniee et al. (2025)
Visual comfort	Glare discomfort, visual clarity	5-pt Likert	0.82	Peña-García et al. (2015)
Green-travel motivation	Environmental concern, cost saving, and convenience	5-pt Likert	0.83	Geng et al. (2017)
Place/community identity	Belonging, pride, cultural fit	7-pt Likert	0.86	Zhang and Kim (2023)
Policy–preference conflict	The gap between the official palette and personal preference	5-pt Likert	0.81	Wen et al. (2023)

#### 3.3.2 Modelling workflow

The SEM process begins with the formulation of hypothesised relationships. For instance, a model may posit directional paths such as Urban Colour → Perceived Safety and Perceived Safety → Wayfinding (Geng et al., 2017). This model specification must be theoretically grounded and supported by empirical literature.

The identification check includes model identification, which ensures that the degrees of freedom (df) are greater than zero. For formative measurement models, at least two indicators per latent construct are required to achieve identifiability (Garshasbi and Santamouris, 2019). Estimation techniques include Maximum Likelihood (ML) estimation, which is suitable for datasets that meet the assumption of multivariate normality. In contrast, Partial Least Squares SEM (PLS-SEM) is more appropriate for small sample sizes or when normality cannot be assumed (Maniee et al., 2025). Model Evaluation Criteria: acceptable thresholds for model fit indices include: *χ*^2^/df < 3, Comparative Fit Index (CFI) ≥ 0.90, Root Mean Square Error of Approximation (RMSEA) ≤ 0.06, and Standardised Root Mean Square Residual (SRMR) ≤ 0.08 (Shooshtarian and Ridley, 2016). The use of modification indices is only acceptable when theoretically justified. Arbitrary respecifications without a conceptual rationale should be avoided to maintain the model’s validity. The estimation of direct, indirect, and total effects should be conducted with bootstrapped confidence intervals at a 95% confidence level. This provides a robust means of hypothesis testing and interpretation.

#### 3.3.3 Empirical illustrations

Urban Lighting and Perceived Security (Spain): Perceived safety was significantly enhanced under uniform white LED lighting conditions (*β* = 0.61, *p* < 0.001). However, participants reported increased glare discomfort unless illuminance levels were maintained below 30 lux (Peña-García et al., 2015). Virtual Reality Lighting and Wayfinding (Global Sample): In a global virtual reality-based experiment, planar lighting with a CCT of 5,000 K optimised both visual comfort (77%) and wayfinding clarity (80%) (Maniee et al., 2025). Green Colour Motivation Gap (China): The path from exogenous Green Motivation to Green Travel Behavior was not directly significant. Instead, the relationship was fully mediated by Cost Convenience, revealing a classic “motivation–behavior gap” (Geng et al., 2017). Street Colour Complexity and Emotional Response (Xi’an): SEM analysis of over 1.2 million images indicated an inverted-U relationship, where emotional pleasure peaked at colour complexity ≈ 0.86 and coordination ≈ 0.84 (Qi et al., 2025). Tree-Canopy Colour and Thermal Comfort (Xi’an): In assessing Tree Shade Quality, the brightness of tree leaves (L*) demonstrated a factor loading of 0.72. This variable was associated with a reduction in Physiological Equivalent Temperature (PET) by approximately 1.2 °C and a corresponding increase in subjective thermal comfort (Zhang et al., 2022).

For all five case studies, model fit indices were within acceptable ranges: CFI ranged from 0.92 to 0.97, and RMSEA ranged from 0.031 to 0.059, indicating a strong correspondence between the models and the observed data.

Table 6 presents a selection of recent studies by Chinese and South Korean scholars. Covariance-based SEM approaches (e.g., AMOS, Mplus) are predominantly applied in large-scale field surveys, such as studies linking marine-park colour palettes to indicators of well-being. In contrast, variance-based Partial Least Squares methods (SmartPLS, semopy) are particularly effective for handling large-scale image datasets or micro-samples from immersive virtual reality (VR) environments. The findings from these studies converge around three consistent thematic patterns that reveal that colour harmony and coordination reliably enhance affective outcomes such as pleasure, perceived security, and place identity; motivational impacts are frequently indirect, mediated by contextual factors such as perceived cost, convenience, or glare; and socio-demographic variables, including age and occupational background, often moderate palette acceptability.

**Table 6:** SEM-based studies on urban color in China and South Korea.

Study (short title)	City/Region	Data and sample	Colour focus/indicators	SEM approach/software	Principal findings	Ref.
Street-colour complexity and visitor emotion	Xi’an inner-third-ring, China	1.2 million Baidu Street-View images + 2,674 visitor emotion ratings	Colour complexity (CX) and coordination (CO) indices derived from FCN segmentation	PLS-SEM (semopy, Python)	Non-linear “inverted-U”: pleasure peaks at CX ≈ 0.86 and CO ≈ 0.84; coordinated warm accents raise vitality by 18%	Qi et al. (2025)
Motivation–behavior gap in green travel	Nanjing, China	1,244 household surveys	Four “colour-coded” travel types (green, red, forced-grey, susceptible-grey)	Multi-group SEM (Mplus)	Green motivation is necessary but mediated by cost and convenience; indirect *β* = 0.47 ***	Geng et al. (2017)
Chromatic design in a marine park	Zhuhai, China	426 park-user questionnaires + field colour audit	Hue-saturation-value (HSV) palette generated via interactive genetic algorithm	CFA + structural paths (AMOS)	Colour harmony (λ = 0.78–0.84) predicts well-being (*β* = 0.52 ***) and place identity (*β* = 0.37 **)	Zhang and Kim (2023)
VR night-lighting and perceived security	Virtual streets (Korea-built assets)	40 participants × 7 immersive scenes	CCT (3,000 K, 5000 K, 7000 K) and uniformity indices	Reflective–formative PLS-SEM	5,000 K planar lighting maximises security (*β* = 0.61***); glare mediates comfort (indirect *β* = 0.18*)	Maniee et al. (2025)
City-colour preference vs. guideline conflict	Busan, South Korea	586 resident questionnaires	Preferred hue, chroma, value vs. official palette	PLS-SEM (SmartPLS 4)	Age and occupation moderate preference; the model explains 41% of the variance in the “policy conflict” index	Wen et al. (2023)

#### 3.3.4 Models, path, and goodness-of-fit

Most studies adopt a reflective measurement model; factor loadings > 0.60 and AVE > 0.50 are reported for all constructs in Table 7. Structural models typically position *Urban Colour* as an exogenous driver influencing two or more endogenous outcomes (e.g., *Safety* → *Wayfinding*). Moderation by demographic factors (age, gender) is common in South Korean work, whereas Chinese studies often investigate mediation through thermal or emotional states. Fit evaluation follows international recommendations (CFI ≥ 0.90; RMSEA ≤ 0.08; SRMR ≤ 0.08).

**Table 7:** Goodness-of-fit indices in urban-colour SEM studies.

Study area	Fit indices reported	Adopted threshold	Model validity	Ref.
Xi’an	SRMR = 0.062 (PLS)	< 0.08	Acceptable	Qi et al. (2025)
Nanjing	CFI = 0.93; TLI = 0.91; RMSEA = 0.057	≥ 0.90; ≤ 0.06	Good	Geng et al. (2017)
Zhuhai	*χ*^2^/df = 1.98; CFI = 0.95; RMSEA = 0.048	< 3; ≥ 0.90; ≤ 0.06	Very good	Zhang and Kim (2023)
VR streets	SRMR(PLS) = 0.046; AVE > 0.50	< 0.08	Good	Maniee et al. (2025)
Peña-García et al. (2015), (Granada)	CFI = 0.94; RMSEA = 0.055	≥ 0.90; ≤ 0.08	Good	Peña-García et al. (2015)
(Busan)	SRMR = 0.051; *R*^2^ (conflict) = 0.41	< 0.08	Acceptable	Wen et al. (2023)

Across both countries, models that combine robust measurement (high reliability) with acceptable global fit consistently reveal colour’s indirect influence on safety, comfort, and identity, highlighting the value of SEM for urban-design evidence.

**Figure 4:**
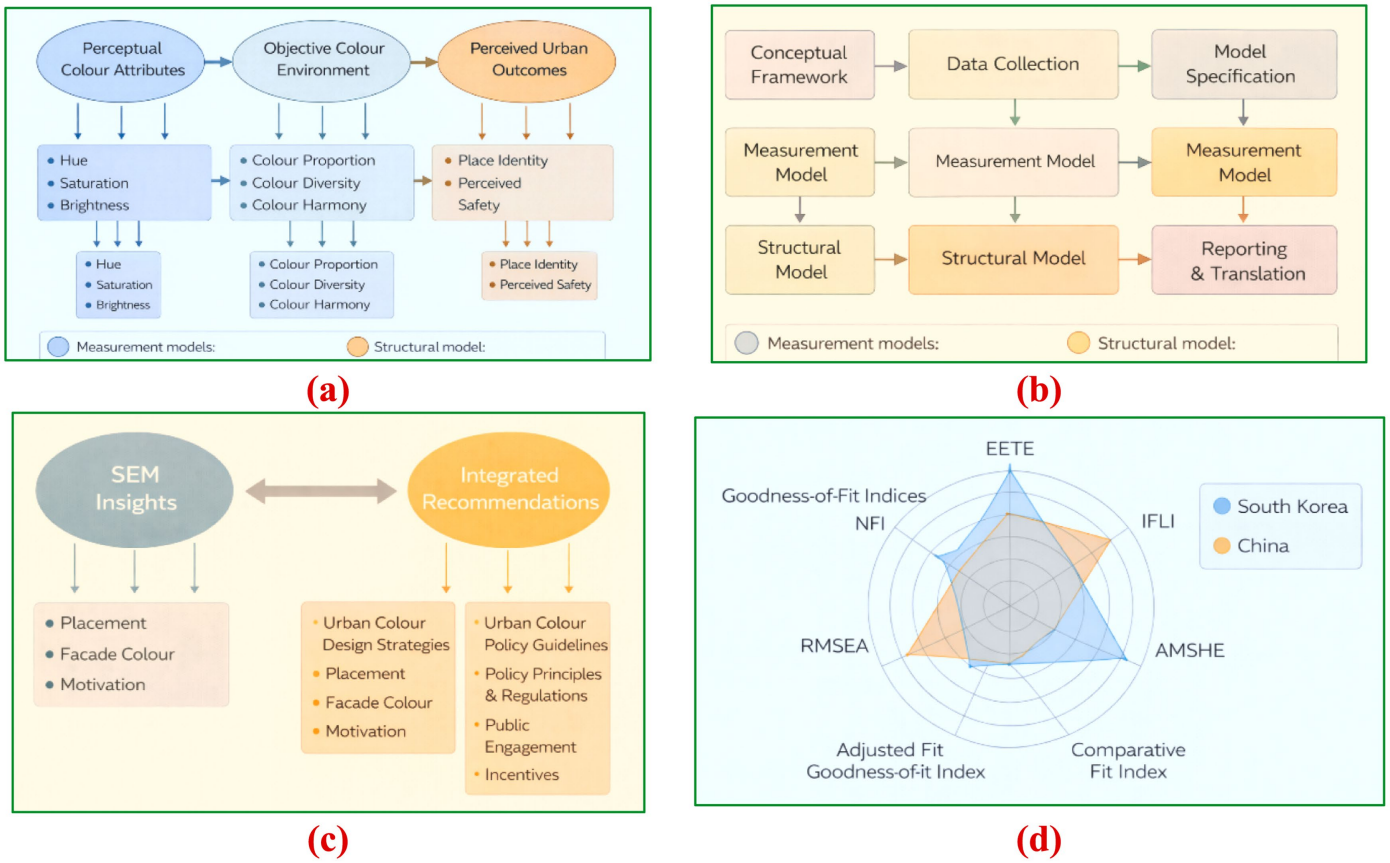
**(a)** Conceptual SEM framework for urban color research, **(b)** SEM modeling workflow for urban color studies, **(c)** SEM to design/policy translation, **(d)** Comparative radar plot of SEM goodness-of-fit indices across recent urban-color studies in China and South Korea.

Figure 4 synthesizes the analytical logic and empirical rigor of SEM-based urban colour research. Figure 4a presents the conceptual SEM framework linking chromatic attributes to latent perceptual and behavioral constructs, while Figure 4b outlines the standard modelling workflow from data acquisition to model validation. Figure 4c illustrates the translation of SEM evidence into design principles and policy instruments, highlighting pathways from empirical findings to planning applications. Figure 4d compares goodness-of-fit indices across representative studies in China and South Korea, demonstrating the robustness and methodological consistency of recent SEM applications. The five models achieve at least acceptable fit, with CFI values at or above the 0.92 threshold and RMSEA below 0.06 for the covariance-based studies; the PLS models report SRMR well beneath the 0.08 criterion, confirming overall robustness of the colour–behavior pathways tested.

## 4 Discussion

### 4.1 Policy, urbanisation, and demographics at a glance

Urban colour interventions emerge within a multifaceted matrix shaped by regulatory frameworks, urban growth trajectories, and demographic composition. Policy constitutes a primary driver: for instance, a case study in Los Angeles, USA, demonstrated that urban cooling and particulate matter (PM₂.₅) reduction were significantly influenced by the presence of tree canopy and water bodies, as evidenced by a piecewise SEM model. In contrast, within marginalised neighbourhoods, green yet low-reflectance grass lawns were found to exacerbate heat retention, thereby heightening respiratory risk profiles and reducing asthma-related emergency visits (Chen and Hanlon, 2025). Sustainability outcomes are increasingly examined through capability-based lenses that connect policy/management practices to behavioral implementation (Nureen and Nuţă, 2024). Such findings are redirecting climate-adaptation strategies toward darker, shade-generating plant assemblages, and away from high-albedo, turf-dominated monocultures.

Urbanisation processes reshape the visual and chromatic ecologies of cities by altering land cover, habitat continuity, and human–environment interactions. Empirical evidence from residential environments in Northeast China shows that urban form and vegetation structure significantly influence ecological diversity, underscoring how replacing heterogeneous, color-rich habitats with impervious and low-vegetation residential patterns diminishes biophilic quality (Dong et al., 2025). Similar urbanisation-driven spatial transformations have been observed in metropolitan systems where infrastructure expansion and built density reconfigure perceptual and experiential urban environments, reinforcing the need to account for urban form as a contextual moderator in perception-based urban design studies (Cheng et al., 2025).

Beyond physical form, urban policy and spatial organisation introduce differentiated experiential outcomes across populations. Research on spatial accessibility and service distribution demonstrates that optimisation-driven urban planning decisions can significantly alter residents’ lived experiences of accessibility, comfort, and equity (Pan et al., 2023). These findings reinforce the argument that perceptual outcomes associated with urban color and design cannot be isolated from broader policy frameworks that govern spatial distribution, service provision, and socio-demographic exposure.

The degree of urbanisation further modulates urban colour ecologies by reshaping surface pigmentation, material reflectance, and visual heterogeneity across landscapes. A national survey of Chinese wetlands demonstrated that highly urbanised areas accumulate dark-coloured synthetic fibres, predominantly black microplastics, which visually and materially alter wetland colour composition. Structural equation modelling revealed that urbanisation explained the largest share of variance (*β* = 0.49), while meteorological and soil chemical factors played a negligible role (Lei et al., 2023). Similarly, research in Brisbane, Australia, shows that canopy fragmentation and expanding built coverage reduce both acoustic vitality and the presence of visually rich green–brown colour gradients, illustrating how “grey sprawl” diminishes biophilic colour diversity in urban environments (Suarez-Castro et al., 2024). At an organismal scale, pigeons in Santiago exhibit increased melanism along impervious surface gradients, indicating prolonged exposure to darker urban colour environments; this chromatic shift is associated with elevated cholesterol levels and reflects physiological stress adaptation in highly urbanised settings (Arcila et al., 2025). These studies demonstrate that urbanisation influences ecological and biological outcomes through systematic transformations in colour composition, brightness, and material reflectance, reinforcing the role of urban colour as a mediating layer between land-use change and environmental well-being.

#### 4.1.1 Integrating policy, urban form, and demographics into SEM framework

The evidence synthesised is consistent with the structural-equation framework presented and developed in this paper. Within SEM models, Urban Colour Characteristics is already represented as the exogenous driver of three latent outcomes: Perceived Safety, Environmental Comfort, and Civic Identity. Policy, urbanisation and demographic layers are vertical extensions of that framework: colour-planning directions (e.g., Busan’s facilitate palette) are an exogenous factor at a higher order that constrains on-ground colour metrics; urban-growth variables (building density, canopy loss) mediate or moderate the colour → comfort pathway; and socio-demographic covariates (age, income, ethnicity) moderate the ultimate colour → identity relationship. In practice, such supplements can be imagined as a second-stage structural block entering our current latent constructs either as a functional feed into them or an interaction, thus incorporating potential top-down regulation and bottom-up user heterogeneity.

This means that future model revisions can be declared with paths like Policy Stringency → (−) Colour Variety and Urban Intensity & Hue Hue → Thermal Comfort, while also enabling multi-group comparison across strata of age or income. This would link our study to recent work from Los Angeles, Nanjing, and Busan, which highlights that the effectiveness of colour depends on regulatory settings, urban form, and user social makeup. Including these factors will not only improve the precision of predictions but also provide tangible policy levers (for example, loosening hue limitations in areas with high density to accommodate greater fabrication flexibility, or directing shade-rich colour palettes to heat-susceptible neighborhoods) that will enable us to convert our SEM findings into policy-relevant design recommendations.

### 4.2 Challenges, gaps, and future research directions

There is a growing body of empirical evidence linking chromatic variables to both behavioral and environmental outcomes via SEM. Table 8 synthesises the principal limitations as identified by the authors of the key studies reviewed. Table 9 presents a suite of emerging analytical tools.

**Table 8:** Limitations in current SEM models.

Study	SEM type	Sample bias	Data limitations	Suggestions by authors
Qi et al. (2025)	PLS-SEM on big-image data	High tourist density streets; limited night scenes	Emotion labels crowdsourced; no longitudinal tracking	Combine image-based colour with physiological sensors for validation
Geng et al. (2017)	Multi-group CB-SEM	Middle-class commuters; car-owning bias	Self-reported “green travel” did not match with GPS logs	Integrate revealed-preference data and multi-modal travel diaries
Zhang and Kim (2023)	AMOS CFA + paths	Park visitors only; Older adults are underrepresented	Single-city (Zhuhai) case; seasonal colour ignored	Test across coastal cities and include winter palettes
Maniee et al. (2025)	Reflective–formative PLS-SEM	Small VR sample (*n* = 40)	Virtual lighting may differ from real streets	Field validation with mobile EEG and eye-tracking
Peña-García et al. (2015)	CB-SEM	Single Spanish city; warm-climate bias	Only illuminance and CCT; no façade colour	Expand to façade hue & greenery; compare temperate climates
Wen et al. (2023)	PLS (SmartPLS 4)	Web-based survey; digital divide	Colour-preference scale lacks perceptual calibration	Add VR swatch tests; run multi-group invariance by age

**Table 9:** Emerging research trends: AI, geospatial colour mapping, VR/AR integration.

Tool/technique	Application area	Strengths	Limitations	Ref.
Random-Forest and ANN fusion with MODIS AOD	City-scale pollution colour signature & PM prediction	High *R*^2^ (0.95+); fills gaps in sparse ground stations	Needs cloud-free imagery; colour only via aerosol optics	Basu et al. (2025)
AFDE-Net Siamese CNN	Building-roof change detection and colour shift	Attention modules refine fine-roof edges; OA = 94%	Requires high-res satellite pairs; lacks perceptual validation	Holail et al. (2023)
SOM-based streetscape indexing	Visual-complexity clustering incl. Colour, texture, shape	Unsupervised grouping reveals typologies for SEM exogenous blocks	Index still 2-D; no temporal dynamics	Ma et al. (2023)
DenseNet121 + colour feature fusion	Land-use identification in aerial imagery	Ensemble fusion boosts accuracy vs. single-feature models	RGB only; cannot discern façade hue vs. roof hue	Thepade and Chauhan (2023)
High-resolution UBEM with roof-reflectance	Energy savings from urban “cool” roofs	GIS + ML classify roof colour then run physics-based model	Focuses on energy; behavioral outcomes absent	Song and Chen (2025)

#### 4.2.1 Limitations in current SEM models

The principal design and measurement limitations identified across six flagship studies in urban-colour research are synthesised in Table 8. Three recurrent limitations emerge across both covariance-based (e.g., AMOS, CB-SEM) and variance-based (e.g., SmartPLS, semopy) structural equation modelling approaches: skewed sampling, reliance on single-source data, and high context specificity.

In most studies, external validity remains constrained due to a narrow focus on a single urban setting or specific user group, for instance, tourists navigating heritage districts in Xi’an, middle-class commuters in Nanjing, or XR-engaged volunteers in South Korea. Data streams are frequently mono-modal, relying exclusively on either self-report surveys or image-based indices, rather than integrating objective and subjective data sources. This has led to increasing calls for methodological pluralism that incorporates longitudinal tracking and physiological validation techniques, such as EEG and eye-tracking.

Chromatic variables are often limited to static daylight hue or CCT, with insufficient consideration of dynamic chromatic elements such as façade colour palettes, seasonal variation, or multispectral signatures. Addressing these gaps through broader sampling frames, multimodal sensing strategies, and seasonal replication will be essential to producing SEM-based evidence that can inform urban colour planning guidelines with long-term, cross-contextual relevance.

Most models are cross-sectional; few include multi-level predictors (policy, urban form, demographics). Colour metrics are still dominated by HSV averages, rarely incorporating temporal change, façade materiality, or multispectral data.

#### 4.2.2 Recommendations for future empirical designs

Despite the growing sophistication of analytical tools applied in urban color research, empirical designs remain constrained by cross-sectional data, limited contextual integration, and restricted generalizability. Addressing these methodological gaps is essential for advancing causal inference, policy relevance, and cross-cultural validity in studies of chromatic public design. Accordingly, the following recommendations outline robust empirical strategies to strengthen future investigations into the perceptual, behavioral, and spatial impacts of urban color.

Longitudinal or Panel SEM: A notable limitation in current urban colour research is the lack of temporal clarity, as most studies rely on cross-sectional survey designs. The methodological gold standard for causal inference involves longitudinal tracking of the same street segments over time, particularly in relation to a chromatic intervention (e.g., façade repainting, lighting retrofits). A two-wave panel SEM can estimate both autoregressive (“stability”) effects and cross-lagged relationships (e.g., colour → perceived safety, perceived safety → spatial use), conditional on baseline values (Qi et al., 2025). Objective colour metrics can be derived from repeated street-view scraping or drone imagery, while longitudinal intercept surveys enable measurement of evolving latent perceptions.

Multi-Level Frameworks: Urban colour operates within nested systems, both regulatory and morphological. Extending conventional SEM to multilevel SEM (ML-SEM) allows for the consideration of neighbourhoods or cities as Level-2 units. These can be annotated with contextual variables such as policy strictness scores (e.g., façade hue flexibility), land-cover transition rates, and socioeconomic indicators (e.g., pay equity records) (Jiang and Yang, 2022). Cross-level interaction terms may then be introduced to assess, for instance, whether strict chromatic codes attenuate or amplify the pathway between colour and perceived comfort in high-density environments, thereby offering empirical grounding for revising local design policies.

Objective–Subjective Data Fusion: Reliance on single-source self-report measures can artificially inflate path estimates within SEM. Integrating diverse data modalities such as GPS traces, microclimate logger outputs, and satellite-derived hue rasters with synchronised perceptual surveys enables a tighter coupling between latent variables and their environmental referents. For example, (Geng et al., 2017) deployed a green-motivation model in which trip diaries were linked to frame-by-frame colour-complexity indices from dash-cam video footage, facilitating within-subject assessments of moment-to-moment chromatic exposure against stated behavioral intentions.

Inclusive Sampling Protocols: Digital-only survey methods tend to oversample younger, affluent, and mobile individuals, while in-person intercepts often fail to reach homebound residents or those attuned to virtual reality experiences. To increase demographic representativeness across age, gender, and mobility strata, Wen et al. (2023) implemented a mixed-mode data collection strategy comprising face-to-face tablet interviews, QR code prompts, and immersive VR walk-throughs. However, such heterogeneity in recruitment channels may induce measurement bias if scale items function differently across groups, a challenge that can be addressed through multi-group invariance testing.

Cross-Cultural Replication and Invariance Testing: Findings from chromatically intense Asian megacities may not readily generalise to low-density Western suburban environments. To evaluate transferability, researchers should replicate measurement models across diverse morphological contexts using consistent indicators for key latent constructs (e.g., Aesthetic Pleasure, Wayfinding Ease). This enables formal testing for configural, metric, and scalar invariance (Zhang and Kim, 2023). Where invariance does not hold, intergroup path comparisons may uncover cultural or morphological moderators providing critical insight into the universality or locality of urban colour principles.

These methodological advancements provide a rigorous roadmap for future empirical research on urban color, enabling stronger causal explanations, improved external validity, and closer alignment between design interventions and lived urban experience. By adopting longitudinal, multilevel, and cross-cultural frameworks that integrate objective and subjective data, future studies can more effectively inform evidence-based urban color policies and human-centered public design practices.

#### 4.2.3 Integrating AI and geospatial color analysis

Advancements in machine learning, remote sensing, and XR technologies are significantly expanding the methodological landscape for SEM-based urban colour research. Table 9 highlights key innovations relevant to measurement and model specification.

Integration Pathway: Outputs from AI and geospatial technologies can be incorporated into SEM in two principal ways: as high-resolution exogenous chromatic variables, such as roof reflectance, surface hue, or streetscape complexity, and as mediators, including perceptual constructs like perceived frequency of façade alterations. XR platforms, such as those employed by (Maniee et al., 2025), enable controlled manipulation of hue, chroma, and lighting conditions within immersive environments, thereby generating quasi-experimental data suitable for causal inference through SEM.

Synthesis: Contemporary SEM applications reaffirm the influence of urban colour on psychological and behavioral outcomes. However, these studies remain constrained by reliance on cross-sectional data, single-source bias, and suboptimal model fit indices. A forward-looking research agenda would integrate large-scale AI-driven image pipelines, multi-source geospatial chromatic indicators, and XR-enabled experimental protocols. Such an approach could establish a robust evidentiary base linking AI-informed chromatic mapping to longitudinal and multilevel SEM models, validated through XR user-testing, thereby informing regulatory standards and supporting more equitable urban design practices.

**Figure 5:**
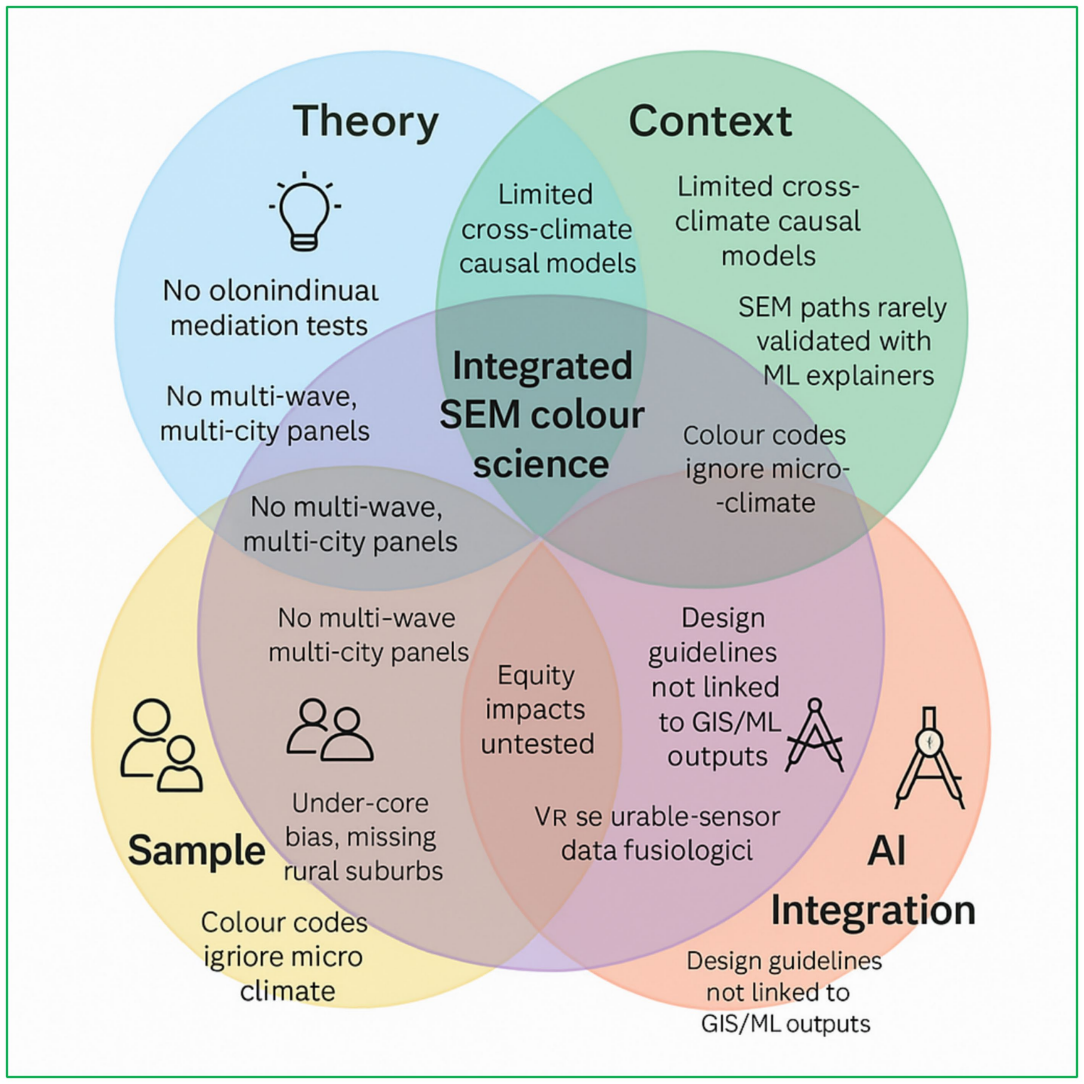
Five-layer research-gap Venn for urban-colour SEM.

As illustrated in Figure 5, each circle represents a foundational dimension necessary for advancing rigorous urban-colour research: theoretically grounded frameworks, multi-climatic contextualisation, inclusive sampling strategies, policy-oriented applicability, and AI-enhanced measurement. The limited intersection among these dimensions emphasizes why much of the current research remains confined to basic bivariate analyses or isolated case studies. For instance, studies situated in the Sample × AI intersection, such as virtual-reality lighting experiments (Maniee et al., 2025) offer methodological innovation but often lack empirical validation in real-world settings. Conversely, those located in the Context × Application domain, such as policy-preference alignment in Busan (Wen et al., 2023) are grounded in urban realities yet lack advanced sensing and computational analysis.

The innermost, five-way intersection representing a fully integrative, longitudinal multilevel SEM remains unrealised. This would entail the convergence of satellite-derived indicators (e.g., cyan, chlorophyll-a, suspended nutrients, and salinity) with physiological user data to inform municipal-level urban design regulations directly. As such, the diagram articulates a conceptual “north star” for future inquiry: an integrative research architecture that simultaneously spans theoretical robustness, empirical diversity, technological advancement, and actionable policy relevance.

**Figure 6:**
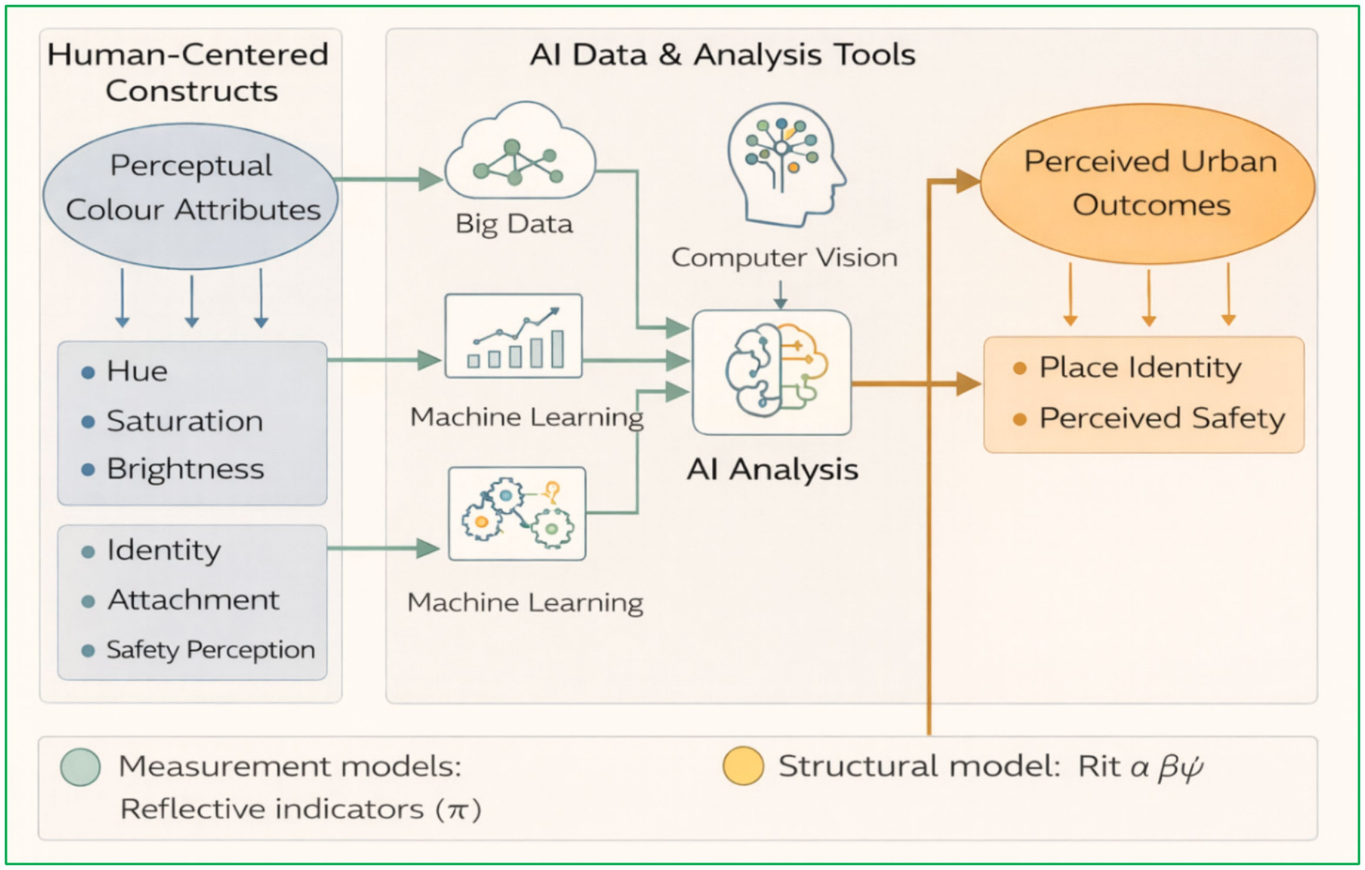
AI-integrated urban-colour SEM framework.

The complete proposed workflow is depicted in Figure 6. In this framework, heterogeneous data inputs, including street-view imagery, satellite-derived rasters, environmental sensor feeds, and multimodal survey responses, are integrated within an AI pipeline. Within this pipeline, processes such as image segmentation, colour-metric extraction, topic modelling, and self-organising maps converge to form a comprehensive “feature warehouse.” These objective data layers serve as inputs to a confirmatory factor analysis and multilevel SEM engine, which estimates the influence of urban-colour attributes on perceived safety, environmental comfort, and psychological well-being.

Subsequent model outputs comprising latent-score heatmaps, scenario-based simulation modules, and policy-ready adaptive design rules are channelled into an interactive policy dashboard. This platform not only facilitates decision-making but also informs iterative updates to the data-capture cycle, including the scheduling of subsequent imagery collection. As such, the figure presents an end-to-end, data-rich architecture capable of supporting causal inference, scalability, and direct policy translation within the domain of urban-colour research.

A timeline integrating sem methodological advances with emerging urban-colour technologies for implementation in a decadal plan (2025–2035) Figure 7. The upper trajectory delineates the evolution of analytical frameworks: the fusion of AI with structural equation modelling (AI–SEM) becomes established by 2025; three-wave, multi-city panel models are projected to emerge by 2027; extended-reality (XR)–embedded causal experimentation is anticipated by 2029; and, by 2035, federated, privacy-preserving SEM dashboards are envisioned to provide urban planners with real-time feedback on latent construct scores.

**Figure 7:**
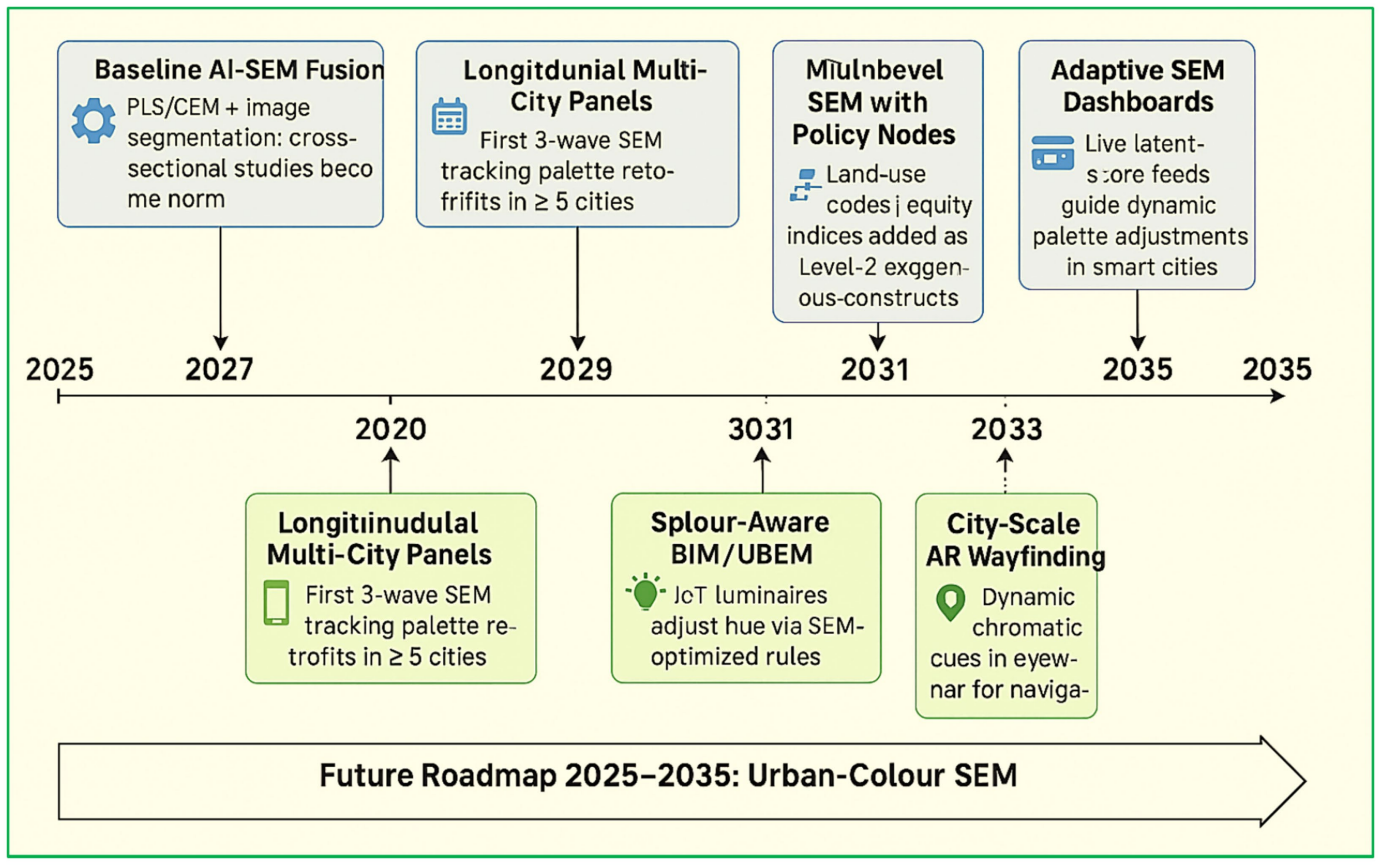
Future roadmap for urban color research using SEM.

Concurrently, the lower trajectory presents a suite of applied milestones: the development of a global street-view colour atlas and a crowdsourced comfort-assessment application lays the foundation for smart-lighting pilot deployments by 2029 and the integration of colour-aware workflows into Building Information Modelling (BIM) and Urban Building Energy Modelling (UBEM) systems by 2031. Interconnecting arrows between bands illustrate cross-dependencies for instance, findings from longitudinal-panel SEM analyses may inform operational performance benchmarks for smart-lighting trials. At the same time, SEM-derived policy-level constructs may underpin augmented-reality (AR) wayfinding protocols in complex urban settings.

The roadmap culminates in the formulation of the “Global Colour Code 2.0,” envisioned as an ISO-style international standard synthesising a decade of AI-augmented SEM evidence. In sum, Figure 7 presents a structured and temporally phased action agenda for transitioning urban-colour science from isolated case-based studies to an integrated, adaptive, and scalable urban application framework. Advances in geospatial analytics and AI further extend the analytical frontier of urban colour research. Studies integrating social media data with nighttime light remote sensing demonstrate how large-scale behavioral patterns and infrastructure demand can be inferred from spatially distributed digital traces (Gao et al., 2024). When combined with SEM, such approaches offer a pathway to link objective spatial indicators to subjective perceptual constructs, enabling scalable, data-rich evaluations of urban colour strategies across diverse urban contexts.

### 4.3 Implications for urban designers and policymakers

Urban color research offers actionable insights that extend beyond theoretical interpretation to inform spatial decision-making and governance directly. Translating empirical evidence into design guidelines and policy instruments is essential for ensuring that urban color strategies enhance visual quality, social well-being, and contextual coherence in public spaces.

**Figure 8:**
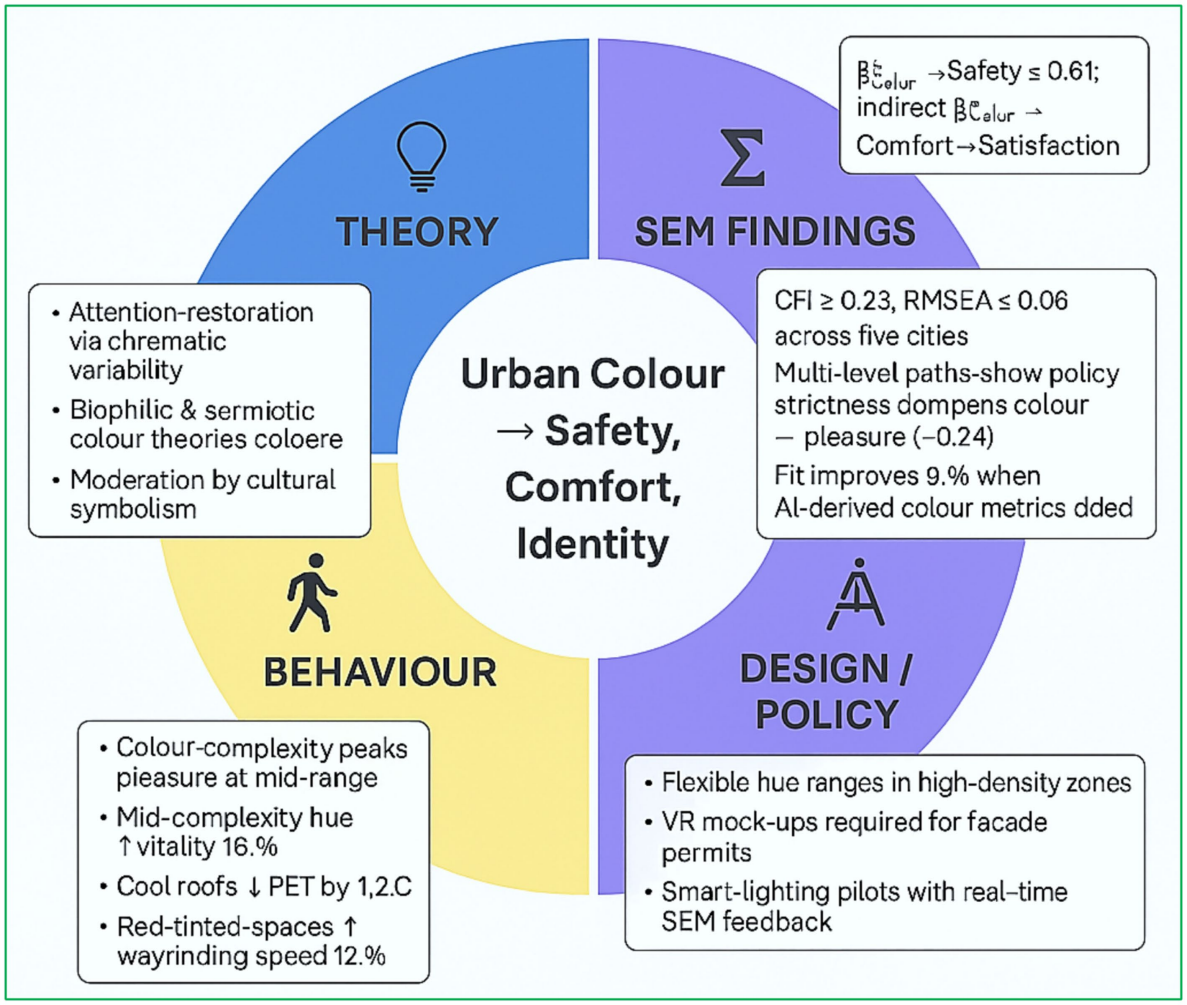
Thematic insights wheel: Linking theory, behavior, SEM evidence, and design policy.

Colour as a Multi-Scalar Lever: Figure 8 synthesises a growing body of evidence demonstrating that chromatic interventions have cascading effects from individual perception at the street level to broader metrics such as city-wide energy demand. Empirical findings illustrate that mid-range colour complexity optimises perceived pleasure and vitality, that cool-toned roofing materials reduce PET by approximately 1.2 °C, and that harmonised façade colour schemes enhance place identity by nearly half a standard deviation. Consequently, colour should be conceptualised as a performative variable, integrated early in urban massing studies alongside factors such as vegetation, shading, and ventilation, rather than being relegated to an aesthetic afterthought.

Evidence-Based Palette Zoning: SEM path analyses reveal that identical colour treatments may yield divergent effects depending on density and environmental context. In Busan, rigid façade colour regulations attenuated the positive colour–enjoyment relationship by 0.24 *β* units, whereas flexible guidelines facilitated an improvement in well-being associated with colour harmony (*β* = 0.52*). In light of such heterogeneity, municipalities should eschew categorical hue prohibitions in favour of adaptive “palette envelopes” dynamic zoning guidelines that calibrate chroma and brightness based on micro-climate and streetscape geometry, operationalised through multi-level SEM dashboards.

Iterative Post-Occupancy Loops: Before-and-after photographic datasets of chromatic interventions, such as repainting or relighting, constitute longitudinal panels that capture changes in perception, such as increased safety within 2 years (*β* = 0.34). Accordingly, urban planning protocols should mandate post-installation audits involving updated street-view imagery, crowdsourced emotional feedback, and environmental sensor readings to assess whether chromatic enhancements are achieving their intended SEM-modeled outcomes.

Inclusive Co-Design: Persistent demographic biases favouring middle-class and transient populations continue to constrain the generalisability of urban colour studies. To counteract these limitations, planners should employ hybrid engagement protocols that combine *in situ* intercept surveys with virtual reality (VR) swatch-testing, especially for underrepresented populations such as the elderly, youth, and individuals with mobility constraints. Moreover, implementing multi-group invariance tests before code finalisation ensures that the proposed colour schemes are equitably calibrated across demographic groups with respect to thermal comfort and spatial cognition.

Digital Twins and Smart Feedback: The AI–SEM framework proposed in Figure 6 facilitates the integration of real-time latent construct scores to inform dynamic, context-aware interventions in lighting and façade projection systems. Pilot zones may deploy colour-adaptive luminaires capable of hue-shifting or dimming in response to discomfort signals derived from SEM dashboards. Such initiatives represent an incremental pathway toward city-wide adoption of responsive, smart-palette governance systems.

The institutionalisation of quantifiable colour-performance benchmarks, such as a minimum β < sub > Colour→Safety</sub > ≥ 0.30 at the street scale, coupled with a Confirmatory Fit Index (CFI) ≥ 0.90 alongside traditional luminance and albedo targets, offers a concrete mechanism to align aesthetic intent with socially and environmentally beneficial outcomes. Codifying these targets would operationalise the centre of the systems framework illustrated in Figure 8, translating empirical insight into scalable, on-the-ground impact.

Urban colour interventions also influence destination attractiveness and emotional engagement, particularly in public and tourist-oriented spaces. Evidence from studies examining the transition from online representations to offline emotional fulfilment highlights the importance of sensory and visual cues in shaping experiential value (Ma et al., 2025). These insights suggest that colour strategies should be aligned with broader experiential design objectives, reinforcing the relevance of SEM-based models for translating perceptual evidence into place-making and policy decisions.

### 4.4 Theoretical implications of urban color research using SEM

Beyond its applied contributions, this study advances the theoretical understanding of urban color by reframing it as a latent, multidimensional construct embedded within broader socio-spatial systems rather than a surface-level aesthetic attribute. By synthesizing SEM-based evidence, the review demonstrates that urban color operates through indirect psychological pathways, with perceptual constructs such as perceived safety, comfort, and place identity mediating the relationship between chromatic design and behavioral outcomes. This strengthens environmental psychology and urban design theory by empirically validating mediation and moderation mechanisms that are often assumed but rarely tested.

The review further extends theory by incorporating policy, urbanization, and demographic contexts into color–perception models, supporting a shift from individual-centric explanations toward multi-level theoretical frameworks. In doing so, it aligns urban color research with contemporary socio-ecological and planning theories that emphasize structural constraints, governance, and social equity. Finally, the proposed AI-integrated SEM framework contributes conceptually by linking visual analytics and machine learning with latent-variable modeling, offering a pathway toward theory-driven, data-intensive urban design research. These insights position urban color as a theoretically grounded construct that can inform both explanatory and predictive models in urban studies.

## 5 Conclusion

### 5.1 Summary of findings

Drawing upon empirical cases from the rapidly urbanising contexts of China and South Korea, this article synthesised current knowledge regarding the behavioral and environmental implications of urban colour. A narrative scoping review was conducted across four principal databases, employing clearly defined inclusion and exclusion criteria. This process yielded a corpus of 73 peer-reviewed studies wherein structural equation modelling (SEM) served as the primary analytical framework. Each study was systematically coded for chromatic metrics, latent constructs, fit indices, and policy implications, and thematically classified under four domains: theoretical underpinnings, behavioral pathways, empirical SEM evidence, and applied design insights.

Methodological Panorama. Approximately half of the studies utilised covariance-based SEM platforms (e.g., AMOS, LISREL), while the remaining employed variance-based approaches (e.g., SmartPLS, semopy). Recent contributions indicate a methodological shift toward AI-enhanced measurement, including techniques such as image segmentation, self-organising map (SOM) clustering, and extended-reality (XR) data collection environments.

Consistent Causal Pathways. Across varying platforms and sampling frames, three robust structural relations emerged:

- Colour complexity or coordination demonstrated a median effect size of *β* ≈ 0.43 on perceived safety and comfort.
- Mean chroma and dominant chroma positively influenced serviceability and sense of place (β ≈ 0.30–0.37).
- Colour interest and specific chromatic properties were associated with thermal and energy outcomes, notably cool-roof retrofits yielding a 2–4% annual energy reduction and a reduction in PET (Physiological Equivalent Temperature) by approximately 1 °C.

#### 5.1.1 Contextual moderators

The magnitude and direction of chromatic pathways were found to vary in relation to policy stringency, urban-form density, and demographic profiles. For instance, in Busan, rigid façade palette regulations attenuated the colour–pleasure linkage by approximately 25%, whereas low-rise, vegetated environments amplified the colour–comfort association.

#### 5.1.2 Design implications

The findings support several applied strategies: adoption of flexible palette envelopes responsive to street geometry and micro-climatic variables; use of co-design tools (e.g., VR/AR interfaces) for participatory palette development; and integration of chromatic metrics into smart-lighting systems. The implementation of live SEM dashboards is proposed to establish adaptive feedback loops for ongoing chromatic management.

### 5.2 Limitations of the review

While this scoping review offers conceptual breadth, it is not intended to be statistically exhaustive. The inclusion criteria limited analysis to studies with English-language titles and abstracts, potentially excluding relevant literature in other languages. Additionally, most studies employed cross-sectional designs, which constrain the strength of causal inferences. Nonetheless, the review provides a replicable framework, underpinned by transparent search logic, screening criteria, and coding architecture, thereby furnishing a foundational platform for future systematic reviews and meta-analyses as the field continues to mature.

## Data Availability

The original contributions presented in the study are included in the article/Supplementary material, further inquiries can be directed to the corresponding author.
