# Supplementary material for: Urban color in public design: a review of spatial aesthetics and behavioral impact in Chinese and South Korean cities using structural equation modelling approaches
Source: Front Psychol. 2026 Jan 27;17:1692740. doi: 10.3389/fpsyg.2026.1692740 (PMC12886025; doi:10.3389/fpsyg.2026.1692740)
Supplement: Supplementary file 2 [file Table_2.DOCX]

## Supplementary Materials

## Structural Equation Modeling (SEM): Basics

### SEM: Type of Data Used in SEM

SEM is a multivariate statistical technique employed to evaluate structural relationships between observed variables and latent constructs. SEM is predominantly applied to quantitative data, with sample sizes typically exceeding 200 to ensure parameter stability and model convergence. Data sources may include surveys, psychological assessments, experimental observations, or longitudinal studies. Frequently, these datasets comprise Likert-scale responses, continuous variables, or other ordinal measurements. SEM is suitable for both cross-sectional and longitudinal data, rendering it particularly applicable to disciplines such as behavioural science, social psychology, urban design, and environmental psychology. The technique generally assumes multivariate normality; however, Partial Least Squares SEM (PLS-SEM) accommodates non-normal data distributions. Before analysis, datasets should be examined for missing values, outliers, linearity, and multicollinearity to ensure robust estimation.

### Steps Involved in SEM

SEM comprises two primary components: (1) the measurement model and (2) the structural model.

Step 1: Model Specification

The researcher defines the hypothesised model based on theoretical foundations and/or empirical evidence. This step involves identifying latent constructs (e.g., satisfaction, trust, safety), their corresponding observed indicators, and the directional relationships among these constructs.

Step 2: Model Identification

This step determines whether sufficient data are available to estimate the model parameters. A model must be overidentified, where the number of known values exceeds the number of parameters to be estimated, for estimation to proceed.

Step 3: Data Preparation

Data cleaning and normalisation are conducted at this stage. Instrument validity (face and content validity) is evaluated, and latent constructs may be preliminarily explored using Exploratory Factor Analysis (EFA), followed by Confirmatory Factor Analysis (CFA).

Step 4: Estimation

Estimation methods include Maximum Likelihood (ML), Generalized Least Squares (GLS), and Bayesian estimation. These techniques generate factor loadings, error terms, and variance estimates.

Step 5: Model Evaluation (Goodness-of-Fit)

Goodness-of-fit indices are employed to evaluate how well the model fits the empirical data. Key indices include:

- Chi-square (χ²)
- Comparative Fit Index (CFI ≥ 0.90)
- Root Mean Square Error of Approximation (RMSEA ≤ 0.08)
- Standardized Root Mean Square Residual (SRMR ≤ 0.08)

Step 6: Model Modification

If model fit is inadequate, modification indices may suggest respecifications, provided that such changes are grounded in theoretical justification.

Step 7: Interpretation

Path coefficients, standard errors, and significance levels are interpreted to evaluate hypothesised relationships. Additionally, mediation and moderation effects may be tested within the model framework.

### Example of SEM Implementation

SEM is commonly implemented through specialised software such as IBM SPSS AMOS, SmartPLS, and the R package lavaan. A typical implementation process using IBM SPSS AMOS is outlined below:

Step 1: Data Preparation

Prepare observed variables in SPSS. Ensure accurate coding and perform reverse coding if necessary.

Step 2: Model Construction in AMOS

Use AMOS Graphics to construct the model via a graphical interface:

- Latent variables are represented by ovals
- Observed variables are denoted by rectangles
- Arrows are drawn to indicate factor loadings and regression paths

Step 3: Parameter Definition and Analysis Execution

Specify the paths and covariances between variables. Click “Calculate Estimates” to run the analysis.

Step 4: Output Analysis

AMOS outputs path coefficients, standardised loadings, and fit indices. Ideal values include:

- CFI > 0.90
- RMSEA < 0.08
- Acceptable χ² fit statistics

### Example Application

Consider a model examining the effect of *Urban Colour* on *Perceived Safety* and *Wayfinding*. Using 12 survey items across three constructs:

- *Urban Colour* (e.g., vividness, brightness)
- *Safety* (e.g., sense of security)
- *Wayfinding* (e.g., ease of navigation)

Paths are specified as:

- Urban Colour → Safety
- Urban Colour → Wayfinding

If the model yields a standardised coefficient of 0.72 (p < 0.001) from Urban Colour to Safety, this indicates a statistically significant and substantively strong relationship.

Alternative Software

- *SmartPLS:* Suitable for small sample sizes or non-normal data; features a user-friendly GUI for PLS-SEM.
- *lavaan (R):* Offers reproducible, script-based SEM analysis for advanced users.

### Example of SEM in Practice

To validate the SEM approach in the context of urban colour, a conceptual model comprising three latent variables, Urban Colour, Perceived Safety, and Wayfinding Behaviour, was constructed. Given its capacity for such integrative analysis, SEM proves highly effective in behavioural and environmental psychology.

In this application, survey data were collected on variables such as colour intensity, street brightness, safety perceptions, and navigation efficiency across various urban environments. The SEM analysis was conducted using the semopy library in Python, which supports flexible model definition, parameter estimation, and fit diagnostics, depicted in Supplementary materials include the complete Python code.

**Step-by-step Python SEM Example: Urban Color → Safety, Wayfinding**

Step 1: Install Required Packages

Use the following pip command to install required libraries:

| pip install semopy pandas numpy |
| --- |

Step 2: Define the SEM Model in semopy Syntax

| from semopy import Model, semplot  import pandas as pd  import numpy as np  # Define SEM model: Latent -> Observed and regression paths  desc = """  UrbanColor =~ vivid + brightness + harmony  Safety =~ secure + safe_env + trust  Wayfinding =~ clear_signs + direction + route_understanding  Safety ~ UrbanColor  Wayfinding ~ UrbanColor  """  # Create dummy dataset (Replace with real data later)  np.random.seed(42)  data = pd.DataFrame({  'vivid': np.random.normal(5, 1, 100),  'brightness': np.random.normal(4, 1, 100),  'harmony': np.random.normal(5, 1, 100),  'secure': np.random.normal(4, 1, 100),  'safe_env': np.random.normal(4.5, 1, 100),  'trust': np.random.normal(5, 1, 100),  'clear_signs': np.random.normal(4.2, 1, 100),  'direction': np.random.normal(4, 1, 100),  'route_understanding': np.random.normal(4.3, 1, 100),  })  # Initialize and fit model  model = Model(desc)  res = model.fit(data)  # Show estimated parameters  estimates = model.inspect()  print(estimates) |
| --- |

Step 3: Evaluate Fit Statistics

| from semopy import calc_stats  stats = calc_stats(model)  print(stats) |
| --- |

**Optional:** Visualize Model (if Graphviz is installed)

| # Optional: visualize SEM graph  semplot(model, "urban_color_sem_model.png") |
| --- |

Note

Replace the dummy dataset with your actual data using:

| data = pd.read_csv('your_file.csv') # or pd.read_excel() |
| --- |

Make sure the column names match the ones used in your model description.
Output:

Path Coefficients from UrbanColor → Safety & Wayfinding

Factor loadings (e.g., vividness → UrbanColor)

Model fit indices (χ², RMSEA, CFI)
